# Water-Tolerant Trifloaluminate Ionic Liquids: New and Unique Lewis Acidic Catalysts for the Synthesis of Chromane

**DOI:** 10.3389/fchem.2018.00535

**Published:** 2018-11-12

**Authors:** Piotr Latos, Alice Culkin, Natalia Barteczko, Sławomir Boncel, Sebastian Jurczyk, Lucy C. Brown, Peter Nockemann, Anna Chrobok, Małgorzata Swadźba-Kwaśny

**Affiliations:** ^1^Department of Organic Chemical Technology and Petrochemistry, Silesian University of Technology, Gliwice, Poland; ^2^The QUILL Research Centre, School of Chemistry and Chemical Engineering, Queen's University Belfast, Belfast, United Kingdom; ^3^Department of Organic Chemistry, Bioorganic Chemistry and Biotechnology, Silesian University of Technology, Gliwice, Poland; ^4^Institute for Engineering of Polymer Materials and Dyes, Gliwice, Poland

**Keywords:** trifloaluminate ionic liquids, metal triflates, chromane, carbon nanotubes, SILP

## Abstract

The first example of triflometallate ionic liquids, named in analogy to chlorometallate ionic liquids, is reported. Trifloaluminate ionic liquids, synthesized from 1-alkyl-3-methylimidazolium triflates and aluminum triflate, were characterized by multinuclear NMR spectroscopy and FT-IR spectroscopy, revealing the existence of oligonuclear, multiply-charged trifloaluminate anions, with multiple bridging triflate modes. Acceptor numbers were determined to quantify their Lewis acidity, rendering trifloaluminate ionic liquids as medium-strength Lewis acids (AN = *ca*. 65). Used as acidic catalysts in the cycloaddition of 2,4-dimethylphenol and isoprene (molar ratio 2:1) to prepare chromane, trifloaluminate systems outperformed literature systems, showing high activity (conversions 94–99%, selectivities 80–89%) and at low loadings (0.2 mol%) at 35°C. Using these new systems as supported ionic liquid phase (SILP) on multi-walled carbon nanotubes (ionic liquid loading 16 wt%) delivered a recyclable catalytic system, with activity enhanced with respect to the homogenous regime.

## Introduction

Lewis acid catalysis is widely used, from multi-ton industrial processes to asymmetric synthesis of fine chemicals. However, traditional Lewis acids (AlCl_3_ and BF_3_) suffer from several drawbacks, in particular high corrosivity and propensity to hydrolysis. Therefore, many Lewis-acid catalyzed reactions must be carried out under strictly anhydrous conditions and using corrosion-resistant vessels. Furthermore, low stability toward water limits application of strong Lewis acids in reactions where water is eliminated, e.g., condensation.

Lewis acid catalysis in organic synthesis is typically carried out in an organic solvent, which comes with a set of challenges in terms of process sustainability, from flammability and issues of recycling/disposal, to difficulties in dissolving both the catalyst and the reactants at sufficient concentrations. Ionic liquids have been used to address some of these challenges: typically inflammable, they also offer enhanced separation and recycling strategies, from liquid biphasic systems to supported ionic liquid phases (SILPs) (Plechkova and Seddon, 2008).

Lewis acidic ionic liquids are nearly synonymous with chlorometallate ionic liquids, i.e., ionic liquids that contain Lewis acidic chlorometallate anions. They are synthesized by the reaction of a metal halide with an organic halide salt (e.g., 1-ethyl-3-methylimidazolium chloride) at various reactant ratios, commonly reported as the molar ratio of the metal halide, χ_MClx_. Lewis acidic systems are typically associated with the excess of metal halide, resulting in the formation of oligonuclear, Lewis acidic anions (Estager et al., 2010, 2014; Hardacre et al., 2010; Atkins et al., 2011). Systems with chloroaluminate(III), chlorogallate(III) or chlorostannate(II) anions may be used to fine-tune the strength of Lewis acidity, and all have been implemented in organic syntheses, prominently in Friedel-Crafts chemistry and Diels-Alder reactions (Markiton et al., 2016; Matuszek et al., 2016). However, although solvent-related issues (flammability, limited solubility) have been addressed, chlorometallate ionic liquids share disadvantages of low stability toward moisture and high corrosivity with their respective metal chlorides. Although some chlorometallate ionic liquids are known as water-stable, most prominently chlorozincate(II) and chloroindate(III) ones (Abbott et al., 2001; Silveira Neto et al., 2004), they too pose corrosion issues due to high chloride content.

An alternative approach to tackling shortcomings of common Lewis acids utilizes Lewis acidic metal trifluoromethanesulfonates (triflates, OTf). Kobayashi and co-workers found that certain Lewis acidic metal triflates (M = Al, Ga, Ln) are stable in aqueous media (Kobayashi and Manabe, 2000; Kobayashi et al., 2002). Following this, such metal triflates were reported as water-tolerant Lewis acidic catalysts for a range of organic transformations: aldol condensations, Diels-Alder reactions, Friedel-Crafts acylations and alkylations, radical additions, aromatic nitrations and sulfonylations (Lub et al., 2005; Coulombel et al., 2009; Robertson and Wu, 2010; Williams and Lawton, 2010; Prakash et al., 2012; Lemière and Duñach, 2013; Markiton et al., 2018). The metal triflate catalysts were active at low concentrations, bearing the promise of cost-effective and efficient processes, but suffered from limitations in solubility. Namely, metal triflates dissolve well only in highly coordinating solvents, such as acetone or acetonitrile, which coordinate to the Lewis acid itself and weaken or disable its catalytic activity, and have very limited solubility in non-coordinating media. Again, ionic liquids have been tested as alternative solvents to overcome the solubility issue: for example, Yb(OTf)_3_ dissolved in 1-butyl-3-methylimidazolium hexafluoroantimonate, [C_4_mim][SbF_6_] at 0.1 M was not only well soluble and catalytically active, but possible recycle and reuse several times (Song et al., 2004; Binnemans, 2007; Sarma and Kumar, 2008; Rao et al., 2011).

In this work, we set out to combine these recent advances in Lewis acid catalysis, in the search of a robust, water-tolerant Lewis acidic catalytic system. Rather than solubilising metal triflates in organic solvents or ionic liquids, a family of triflometallate ionic liquids was synthesized. In analogy to chlorometallate systems, they contained metallate anions, but surrounded by triflate, rather than chloride, ligands. Following a multi-technique speciation study, trifloaluminate systems were used as Lewis acidic catalysts in a model reaction: [3+3]-cycloaddition of 2,4-dimethylphenol and isoprene to prepare chromane. Seeking for the best recycling strategy, catalytic reactions were carried out homogenously, under solventless conditions, and heterogeneously, with trifloaluminate ionic liquids immobilized on the surface of carbon nanotubes (SILP).

## Experimental section

### Materials and methods

All air-sensitive materials were handled under an nitrogen or argon, using standard Schlenk line and glovebox techniques. Solvents were used without purification, or dried by purging with argon. CDCl_3_, CD_3_OD, and C_2_D_6_SO for NMR spectroscopy were purchased from Sigma Aldrich and used without purification.

2,4-Dimethylphenol, isoprene, and triflate salts of Al(III), Sn(II), Sc(III), In(III), Yb(III), Ga(III) Zn(II), La(III), Li(I), Ag(I), Er(III), Bi(III), Y(III), and Tl(III), and nitrobenzene were purchased from Sigma-Aldrich. Multiwalled carbon nanotubes, MWCNTs, were supplied from CheapTubes™ (USA). 1-Ethyl-3-methylimidazolium triflate, [C_2_mim][OTf], and 1-octyl-3-methylimidazolium triflate, [C_8_mim][OTf], were purchased from Sigma-Aldrich.

#### Analytical methods

GC analyses were performed using a SHIMADZU GC-2010 Plus equipped with a Zebron ZB-5MSi column (30 m × 0.32 mm × 0.25 μm film). GC-MS analyses were performed using an Agilent GC 7890C (HP-5 MS capillary column, 30 m × 0.25 mm × 0.25 μm, conjugated with an Agilent mass spectrometer 5975C with EI ionization (70 eV). The products were identified using the NIST/EPA/NIH Mass Spectral Library.

Nitrogen adsorption/desorption isotherms for the carbon materials were obtained using a Micrometrics ASAP 2420M instrument at −196°C to calculate their specific surface areas (SBETs) and pore volumes. The size of the pores was obtained using the Barrett–Joyner–Halenda (BJH) method with the Kruk–Jaroniec–Sayari correction. Prior to the experiments, the samples were out-gassed at 200°C and 1.33·10^−3^ Pa for 5 h.

The ionic liquid loadings on the surface of the CheapTubes™ MWCNTs were determined by thermogravimetry (TGA), using a Mettler-Toledo STAR851 thermobalance. Samples (5–10 mg) were placed in standard 70 μL Al_2_O_3_ crucibles and heated from 25°C to 800°C at a rate of 20°C min^−1^, under nitrogen flow of 100 cm^3^ min^−1^. TG, DTG and DTA curves were recorded (SD, Figures S8-S12).

Infrared spectra of neat ionic liquid samples were recorded on a Perkin Elmer Spectrum 100 Series FT-IR spectrometer with a universal ATR accessory. Eight scans were acquired for each sample.

NMR spectra of ionic liquids were recorded using Bruker AVANCE 400, at the following operating frequencies: ^1^H 399.78 MHz, ^31^P{^1^H} 161.83 MHz, ^19^F 376.17 MHz, ^13^C{^1^H} 100.53 MHz. NMR spectra of 2,4-dimethylphenol, isoprene and chromane were recorded using Agilent 400-MR, at the following operating frequencies: ^1^H 399.89 MHz, ^13^C 150.90 MHz. Chemical shifts are quoted as parts per million.

#### Differential scanning calorimetry (DSC)

All scans were obtained using a TA DSC Q2000 model with a TA Refrigerated Cooling System 90 (RCS) and an autosampler. The samples were sealed in the glovebox in TA Tzero aluminum pans with hermetic lids. The temperature was ramped from −90 to 100°C at 5°C min^−1^, then stabilized at 100°C for 5 min, and subsequently cooled to −90°C at 5°C min^−1^, then stabilized for 5 min, and the whole cycle was repeated two more times; the DSC chamber was filled with dry nitrogen. Curves representative of glass transitions are shown in Figure S6.

#### Acceptor number determination

For each ionic liquid, three samples (*ca*. 1 g each; SD, Tables S1–S2) were weighed out in a glovebox, and mixed with a ^31^P NMR probe molecule, triethylphosphine oxide (TEPO), at different TEPO concentrations (3–10 mol%). After dissolution of TEPO was ensured, the solutions were loaded into NMR tubes, containing sealed capillaries with d6-dimethylsulfoxide (an external lock).

^31^P NMR spectra were recorded at 80°C, at 161.98 MHz, using a Bruker AvanceIII 400 MHz spectrometer. Phosphoric(V) acid, 85% solution in water, was used as an external reference. Three solutions of TEPO in hexane (*ca*. 5, 10 and 15 mol %) were prepared, and then measured at 27, 57, 87°C (Tables S3, S4). For each TEPO-ionic liquid system, the ^31^P NMR chemical shift for the infinite dilution of TEPO, δ_inf_, was determined by extrapolation from the ^31^P NMR chemical shifts measured at different TEPO concentrations. The chemical shift of TEPO in hexane, extrapolated to infinite dilution, δ_inf hex_, was used as a reference (δ_inf hex_ = 0 ppm). The AN values for all samples were calculated from the following formula: AN = 2.348·δ_inf_ (Gutmann, 1978; Estager et al., 2010).

### Synthetic procedures

#### Synthesis of trifloaluminate ionic liquids

In the glove box, [C_2_mim][OTf] or [C_8_mim][OTf] and Al(OTf)_3_ were weighed into a vial at various molar ratios, expressed as a molar fraction of Al(OTf)_3_: χ_Al(OTf)3_ = 0.15, 0.25, 0.33, 0.375, 0.40, 0.50. The reactants were stirred at 85°C for 2 h to homogenize. Samples were prepared at 1 g scale and used without further purification (SD, Tables S3–S4). All ionic liquids were stored in the glovebox until used, and samples for NMR studies of neat ionic liquids and AN measurements were prepared in the glovebox. Typical NMR spectroscopic analyses are given below.

[C_2_mim][OTf]-Al(OTf)_3_, χ_Al(OTf)3_ = 0.15: ^1^H NMR (DMSO, 400 MHz, 360 K): δ 8.05 (s, 1H), 6.87 (s, 1H), 6.81 (s, 1H), 3.58 (m, 2H), 3.26 (s, 3H), 0.83 (m, 3H); ^13^C NMR (DMSO, 400 MHz, 360 K): δ 135.54, 122.97, 121.30, 119.51 (q, ^1^J_CF_ = 318 Hz), 44.15, 34.94, 13.49; ^19^F NMR (DMSO, 400 MHz, 360 K): δ−79.42 (s, -CF_3_); ^27^Al NMR (DMSO, 400 MHz, 360 K): δ−13.63 (s, Al(OTf)_6_).

[C_8_mim][OTf]-Al(OTf)_3_, at χ_Al(OTf)3_ = 0.15: ^1^H NMR (DMSO, 400 MHz, 360 K): δ 8.05 (s, 1H), 6.87 (s, 1H), 6.81 (s, 1H), 3.58 (m, 2H), 3.26 (s, 3H), 0.83 (m, 3H); ^13^C NMR (DMSO, 400 MHz, 360 K): δ 136.05, 123.16, 121.88, 119.77 (q, ^1^J_CF_ = 318.1 Hz, C_13_), 49.21, 35.17, 30.79, 29.06, 28.01, 27.97, 25.26, 21.56, 12.68; ^19^F NMR (DMSO, 400 MHz, 360 K): δ−79.12 (s, -CF_3_),−79.49 (shoulder, Al-OTf); ^27^Al NMR (DMSO, 400 MHz, 360 K): δ−12.56 (s, Al(OTf)_6_).

#### Preparation of immobilized ionic liquids

[C_2_mim][OTf]-Al(OTf)_3_], χ_Al(OTf)3_ = 0.25 (0.200 g), MWCNTs (0.400 g) and hexane (10 cm^3^) were introduced into a 25 cm^3^ round-bottom flask. The flask was sealed with a septum and mixed in an ultrasonic bath (20°C, 2 h). Then, the mixture was filtered through a Büchner funnel, washed with 15 cm^3^ of hexane and dried at 50°C under vacuum (Schlenk line) for 2 h.

#### Synthesis of 2,2-dimethyl-2,4-dimethylchromane

The catalyst: Al(OTf)_3_, trifloaluminate ionic liquids or immobilized trifloaluminate ionic liquids, used at 0.2–2 mol% per isoprene, was added to a stirred solution of 2,4-dimethylphenol (0.489 g, 4 mmol) and isoprene (0.272 g, 2 mmol), at 35°C. Reaction progress was monitored by GC. After completion, *n*-hexane (3 cm^3^) was added to the reaction mixture to separate the catalyst: Al(OTf)_3_ precipitated as white powder, ionic liquids phase-separated from the reaction mixture, and immobilized ionic liquids could be separated by filtration. After separation of the catalyst, the product was isolated as a colorless oil using column chromatography (SiO_2_, *n*-hexane:AcOEt 100:1).

2,2-dimethyl-2,4-dimethylchromane: ^1^H NMR (400 MHz, CDCl_3_, TMS): δ 1.32 (s, 6H), 1.76-1.79 (t, J = 6.8 Hz, 2H), 2.14 (s, 3H), 2.23 (s, 3H), 2.72-2.75 (t, J = 6.8 Hz, 2H), 6.72 (s, 1H), 6.78 (s, 1H). ^13^ C NMR (150 MHz, CDCl_3_): δ 16.07, 20.56, 22.78, 27.19, 33.12, 73.80, 120.10, 126.11, 127.26, 127.98, 129.24, 150.06; GC-MS: (EI) m/z (%) 190 (40, M^+·^), 175 (16), 135 (100), 134 (27), 106 (12), 91 (38), 77 (16), 65 (11), 41 (23), 39 (27).

#### Leaching studies

To test for catalyst leaching from the support, the reaction was stopped after 15 min, the catalyst was filtered off, and the reaction was continued. Results were compared to these without the filtration step.

#### Recycling studies

For recycling experiments, reactions were scaled up by a factor of five. After completion of the reaction, *n*-hexane was added (15 cm^3^) and the catalyst was separated. Ionic liquids phase-separated (0.075 g) and was washed with *n*-hexane (3 × 5 cm^3^). Al(OTf)_3_ was filtered off (0.200 g) and washed with *n*-hexane (3 × 5 cm^3^). When using supported ionic liquids, the addition of *n*-hexane resulted in phase-separation of the second liquid phase (the ionic liquid phase, 0.091 g), which was re-immobilized on the MWCNTs surface using standard procedure. Irrespective of the catalyst, in the final step *n*-hexane was removed from the catalyst (60°C under vacuum on Schlenk line, 1 h), and the catalyst was used in the next cycle of the reaction.

## Results and discussion

### Synthesis and characterization of trifloaluminate ionic liquids

A wide range of Lewis acidic metal triflates are commercially available: the most costly are Sn(OTf)_2_, Sc(OTf)_3_, and Yb(OTf)_3_ (30–70 Euro per 1 g from Sigma-Aldrich), followed by Bi(OTf)_3_, Ga(OTf)_3_, and In(OTf)_3_ (*ca*. 17 Euro per 1 g). Al(OTf)_3_, priced at *ca*. 6 Euro per 1 g, is by far the least costly option. Bearing in mind that the success of chloroaluminate ionic liquids lied in their high Lewis acidity combined with low cost of the metal salt, it was found reasonable to select this least expensive triflate for this study.

**Figure 1:**
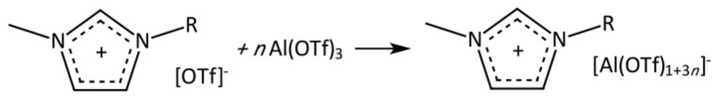
The synthesis of [C_2_mim][OTf]-Al(OTf)_3_.

The first examples of trifloaluminate ionic liquids (Figure 1) were synthesized, by analogy to chloroaluminate systems (Estager et al., 2010, 2014; Hardacre et al., 2010; Atkins et al., 2011)., through a solventless reaction of [C_2_mim][OTf] or [C_8_mim][OTf] with Al(OTf)_3_, at various molar ratios of reactants, expressed as the molar fraction of Al(OTf)_3_: χ_Al(OTf)3_ = 0.15, 0.25, 0.33, 0.375, 0.40, 0.50 (see Tables S1, S2 for experimental details).

Reactions were carried out for 2 h at 85°C, and the products' appearance varied from colorless, homogeneous liquids to pastes consisting of extremely viscous liquids and white solids. This trend, summarized in Table 1, is in stark contrast to chloroaluminate ionic liquid series, where viscosity decreases with increasing loading of AlCl_3_, and which form homogenous liquids up to χ_AlCl3_ = 0.67 (Welton, 1999; Estager et al., 2014). Despite several attempts, it has not been possible to record melting points for either system; in DSC scans all homogenous liquid samples featured weak glass transitions, with onsets between −75.15 and −13.15°C (Figure S6).

**Table 1:** The appearance of trifloaluminates as a function of χ_Al(OTf)3_.

**Entry**	**χ_Al(OTf)3_**	**Physical state**	
		**[C_2_mim][OTf]-Al(OTf)_3_**	**[C_8_mim][OTf]- Al(OTf)_3_**
1	0.15	Pale liquid	Pale liquid
2	0.25	Viscous liquid	Liquid
3	0.33	Viscous liquid with suspended solid	Viscous liquid
4	0.375	–	Very viscous liquid with suspended solid
5	0.40	Very viscous liquid with suspended solid	Very viscous liquid with suspended solid
6	0.50	Glass with suspended solid	Extremely viscous liquid with suspended solid

The investigation concerning the structure of newly synthesized trifloaluminate anions was carried out using different techniques, such as multinuclear NMR, variable temperature NMR and FT-IR spectroscopy. Such difference in physical properties indicates fundamental differences in anionic speciation between trifloaluminate and chloroaluminate ionic liquids. Multi-technique spectroscopic studies were employed to shed light on the speciation of the newly synthesized triflometallate systems.

#### NMR spectroscopy

^27^**Al NMR spectra** are often instrumental in elucidating the nature of aluminate species, with four-coordinate species commonly found at 40 to 140 ppm, five-coordinate at 25–60 ppm, and six-coordinate at −46 to 40 ppm (Olah et al., 1984; Atwood, 1998; Choi et al., 2009; Apblett, 2012; Tayade et al., 2015).

**Figure 2:**
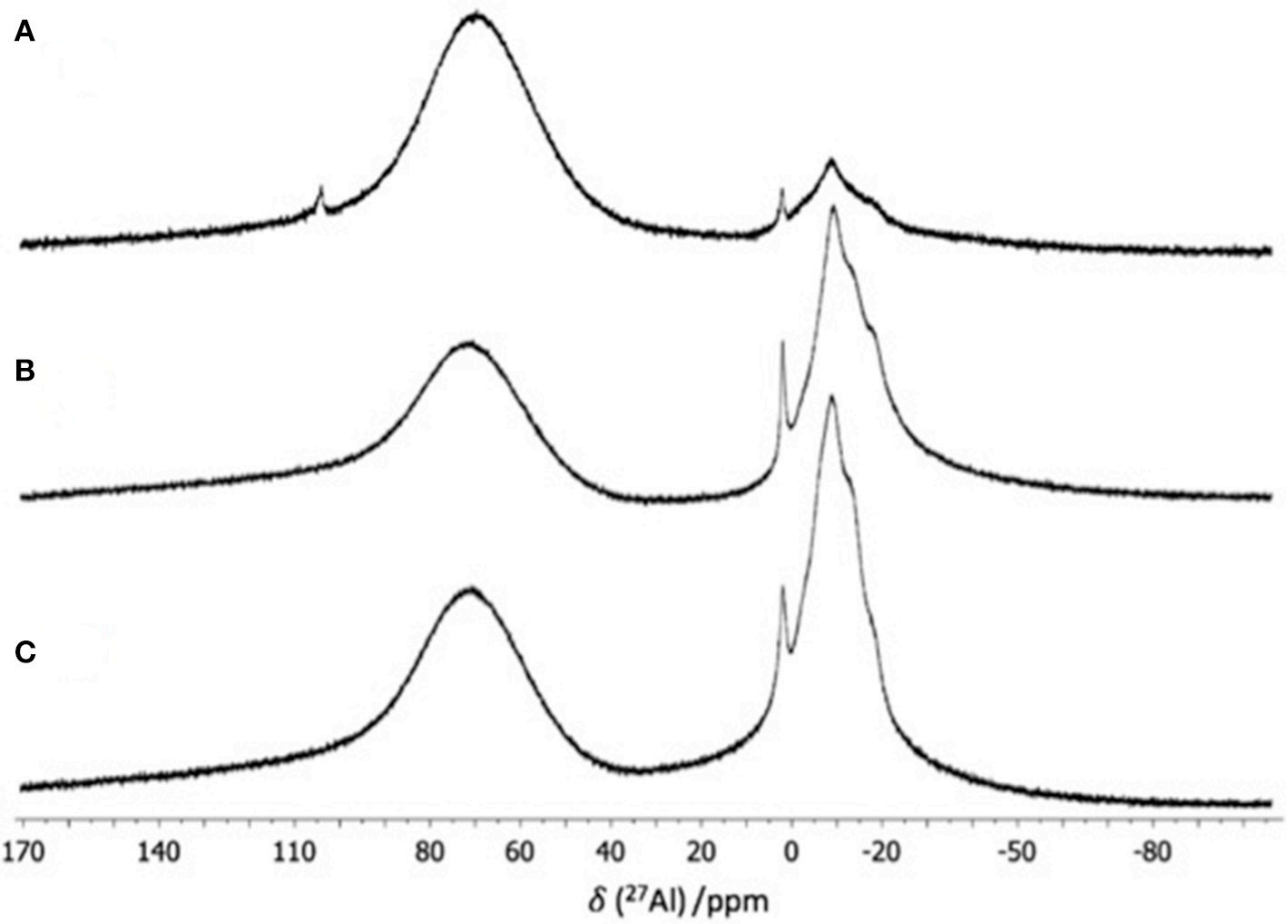
^27^Al NMR spectra of [C_8_mim][OTf]-Al(OTf)_3_, χ_Al(OTf)3_ = **(A)** 0.15, **(B)** 0.25, and **(C)** 0.33 recorded neat, with *d*_6_-dmso capillary, at 57°C).

^27^Al NMR spectra of three homogenous samples of the [C_8_mim][OTf]-Al(OTf)_3_ system (χ_Al(OTf)3_ = 0.15, 0.25 and 0.33), recorded at 27°C, are shown in Figure 2. All spectra feature broad signals, with pronounced NMR probe signal at around 65 ppm. All samples feature a group of signals between−10 and 1 ppm, corresponding to multiple six-coordinate ^27^Al species. Only for χ_Al(OTf)3_ = 0.15, there is a singlet at 103 ppm (four-coordinate ^27^Al), which is rather surprising given high excess of triflate anions in this sample. The prevalence of the six-coordinate species is expected for χ_Al(OTf)3_ = 0.15 and 0.25, considering the excess of triflate ligands per each aluminum atom (around 8:1 and 6:1, respectively). However, mononuclear complexes at χ_Al(OTf)3_ = 0.33 were expected to be five-coordinate, or to comprise a combination of four- and six-coordinate species, given triflate-to-Al ratio of 5:1.

Increasing temperature of NMR measurements may have profound effect on the spectrum: exchange rate between species increases (peaks merge), viscosity decreases (peaks are better resolved), species may appear/disappear. Variable temperature ^27^Al NMR studies (SD, Figures S1–S3) were carried out, with spectra recorded between 27 and 87°C. In all cases, there were differences in shape and resolution of ^27^Al NMR signals, which is unsurprising given temperature-induced changes in viscosity of the neat samples, and changes in rate of exchange between species, but no fundamental alterations in coordination numbers were found. Furthermore, variable temperature ^27^Al NMR studies of the two homogenous, liquid samples of the [C_2_mim][OTf]-Al(OTf)_3_ system (χ_Al(OTf)3_ = 0.15 and 0.25) featured only multiple six-coordinate environments of ^27^Al (SD, Figure S3).

^27^Al NMR studies suggest that aluminum surrounded exclusively by triflate ligands shows strong propensity for six-coordinate environment, in contrast to four-coordinate environment in chloride-rich environment. This is in agreement with multiple crystallographic data, demonstrating that aluminum adopts coordination number of 6 when surrounded by *O*-donors (Finnegan et al., 1986). Multiple signals strongly suggest multiple hexacoordinate complexes, suggesting a variety of triflate coordination modes in aluminum complexes. Furthermore, six-coordinate environment at χ_Al(OTf)3_ = 0.33 indicates the presence of multinuclear species, with triflates in bridging coordination modes.

^19^**F NMR spectra** were recorded for a range of [C_8_mim][OTf]-Al(OTf)_3_ compositions: χ_Al(OTf)3_ = 0.15-0.40, and for aluminum-free triflate ionic liquid, [C_8_mim][OTf] (χ_Al(OTf)3_ = 0.00). Only the liquid part of the χ_Al(OTf)3_ = 0.40 composition was studied.

**Figure 3:**
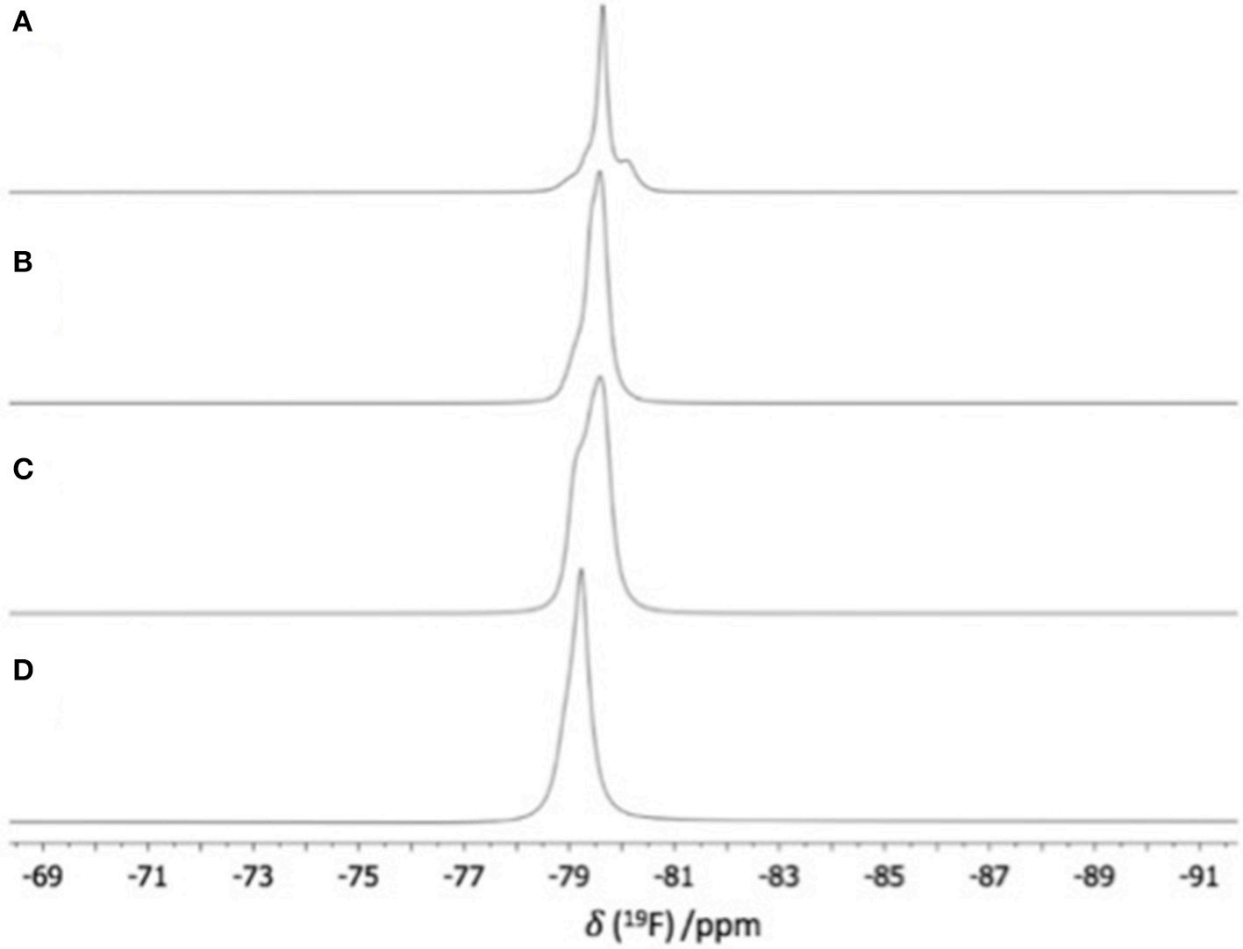
^19^F NMR spectra of [C_8_mim][OTf]-Al(OTf)_3_, χ_Al(OTf)3_ = **(A)** 0.15, **(B)** 0.25, **(C)** 0.33, and **(D)** 0.40 (recorded neat, with *d*_6_-dmso capillary, at 57°C).

As shown in Figure 3, ^19^F NMR spectrum of the neat ionic liquid features a singlet characteristic of free triflate at −80.2 ppm (Burger and Baumeister, 1999) which prevailed in trifloaluminate ionic liquids up to χ_Al(OTf)3_ = 0.33, with significant broadening as the Al(OTf)_3_ loading increased, but was absent from the Al(OTf)_3_-saturated sample, χ_Al(OTf)3_ = 0.40. Upon the addition of Al(OTf)_3_ to [C_8_mim][OTf] several new signals appeared, very slightly deshielded with respect to free triflate (range here −79.83 to−78.93 ppm). Also these signals broadened with increasing Al(OTf)_3_ loading; four peaks noticeable at χ_Al(OTf)3_ = 0.15 merged into two peaks with a shoulder at χ_Al(OTf)3_ = 0.40. Intensity of the singlet originated from the free triflate group decreased with increasing mole fraction of Al(OTf)_3_. The presence of free triflate at χ_Al(OTf)3_ = 0.33 (triflate-to-aluminum ratio 5:1), together with the confirmed coordination number of 6 for all Al species, suggests that aluminum in a triflate-rich environment has the preference toward oligomeric clusters with multiple bridging triflates.

The general peak broadening likely results from the increasing viscosity of neat ionic liquids. Multiple signals from trifloaluminate anions corroborate with ^27^Al NMR spectra, suggesting several coordination modes that triflate ligands adopt. Variable temperature ^19^F NMR studies (SD, Figure S4), with spectra recorded between 27 and 87°C, show that signals merge progressively, to form sharp signals at 87°C, which indicates increased rate of dynamic exchange between free and coordinated triflate.

#### FT-IR spectroscopy

Both [C_8_mim][OTf]-Al(OTf)_3_ and [C_2_mim][OTf]-Al(OTf)_3_ systems were analyzed with FT-IR spectroscopy across compositional range, and compared to the corresponding starting materials. The key triflate absorption were identified: asymmetric S-O stretch *ca*. 1,260 cm^−1^ and asymmetric C-F stretch at *ca*. 1,160 cm^−1^ (Table 2; Gejji et al., 1993; Johnston and Shriver, 1993).

**Table 2:** Vibrational frequencies (cm^−1^) of the key molecular vibrations in triflates, for Al(OTf)_3_, [C_2_mim][OTf] or [C_8_mim][OTf], and their mixtures.

**Assignment**	***χ***_**Al(OTf)3**_ **in [C**_**2**_**mim][OTf]-Al(OTf)**_**3**_				
	**0.00**	**0.15**	**0.25**	**0.50**	**1.00**
ν_SO3 ass_ (cm^−1^)	1252.4	1260.9	1289.1	1302.9	1221.8
ν_CF3 ass_ (cm^−1^)	1154.7	1159.8	1162.8	1166.1	1180.3
	χ_Al(OTf)3_ **in [C**_8_**mim][OTf]-Al(OTf)**_3_				
ν_SO3 ass_ (cm^−1^)	1254.0	1254.8	1287.6	1292.5	1221.8
ν_CF3 ass_ (cm^−1^)	1157.0	1158.6	1163.1	1165.4	1180.3

**Figure 4:**
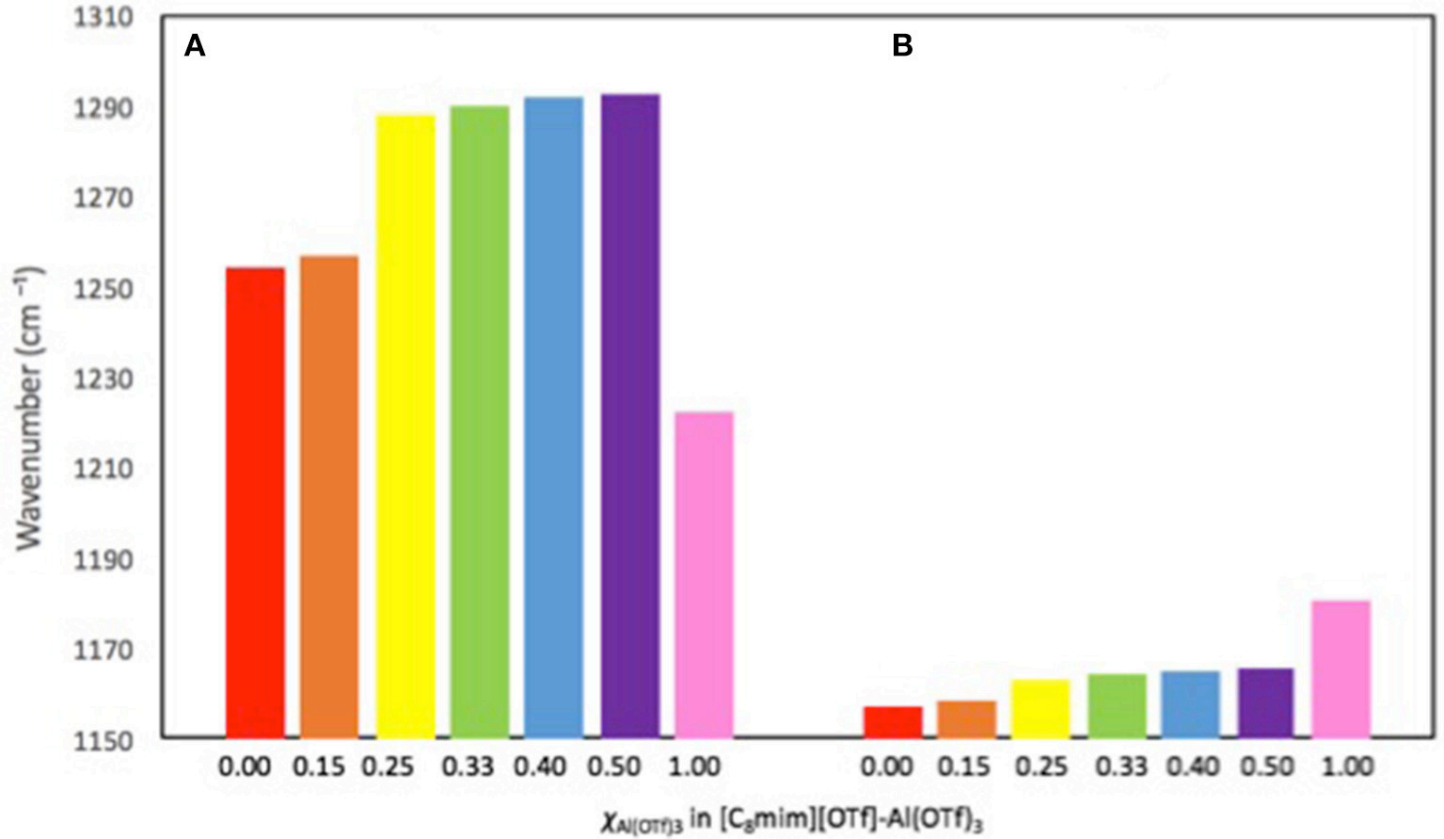
Wavenumbers (cm^−1^) of the characteristic triflate molecular vibrations: **(A)** ν_SO3ass_ and **(B)** ν_CF3ass_ in Al(OTf)_3_, [C_8_mim][OTf], and their mixtures.

Both set of results are similar, and follow the same trend. To facilitate the interpretation, a graphical representation of the results for [C_8_mim][OTf]-Al(OTf)_3_ has been plotted in Figure 4. At a glance, there are three value ranges adopted by both ν_SO3 ass_ and ν_CF3 ass_. Firstly, triflate in Al(OTf)_3_ is characterized by red-shifted S-O vibrations (1,221.8 cm^−1^) and blue-shifted C-F vibrations (1,180.3 cm^−1^). Secondly, free triflate in the ionic liquid is characterized by slightly higher energy S-O vibrations (1,254.0 cm^−1^) and red-shifted C-F vibrations (1,157.0 cm^−1^). Results for very low loading of Al(OTf)_3_, χ_Al(OTf)3_ = 0.15, are the same as for free triflate; it appears that bands for trifloaluminate anions have been obscured by signals from free triflates. Thirdly, all compositions χ_Al(OTf)3_ = 0.25–0.50 (only liquid parts considered) share very similar spectroscopic features, with blue-shifted S-O vibrations (~1,290.5 cm^−1^) and intermediate values for C-F vibrations (~1,164.5 cm^−1^). It can be assumed that the last set of values corresponds to characteristic vibrations of trifloaluminate anions in ionic liquid.

### Discussion on speciation

Multiple signals corresponding to six-coordinate aluminum in ^27^Al NMR spectra, combined with the presence of free triflates up to χ_Al(OTf)3_ = 0.33 (from ^19^F NMR spectroscopy) suggest propensity of aluminum to form a variety of oligomeric complexes with triflates in bridging modes. Viscosity visibly increasing with the increasing χ_Al(OTf)3_ value indicates the formation of multiply-charged species.

In discussing liquid-phase speciation, it is common to refer to relevant solid-state structures; however, these are not available for Al(OTf)_3_, or any trifloaluminate anions. Indeed, no M(OTf)_3_ crystal structures are known. The only single crystal structures recorded by conventional methods are these of Group 1, K(OTf) (Korus and Jansen, 2001) and Na(OTf) (Sofina et al., 2003), in addition to several M(OTf)_2_ structures, recoded relatively recently by synchrotron X-ray powder diffraction data (Dinnebier et al., 2006). All of these feature triflate anions in complex binding modes, both terminal and bridging, with a single oxygen in triflate shared by one, two or three metal centers. In this work, multiple attempts to grow a single crystal suitable for structural studies, from any composition from the [C_2_mim][OTf]-Al(OTf)_3_ system, failed.

Clues provided from above data, both literature and spectroscopic, lead to conclusion that the homonuclear trifloaluminate anions are not the thermodynamically preferred. The nominal stoichiometry for the [Al(OTf)_6_]^3−^ anion is χ_Al(OTf)3_ = 0.25, but this composition was shown to contain free triflate and multiple environments of ^27^Al, indicative of multinuclear species. Furthermore, it has not been possible to obtain a homogenous sample with χ_Al(OTf)3_ = 0.50 composition, where [Al(OTf)_4_]^−^ would be expected in analogy to [AlCl_4_]^−^.

**Figure 5:**
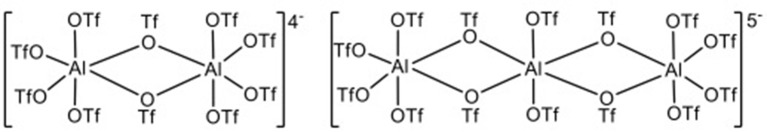
Plausible anionic species present in trifloaluminate ionic liquids, close to the maximum loading of Al(OTf)_3_: [Al_2_(OTf)_10_]^4−^ and [Al_3_(OTf)_14_]^5−^.

The limit for accessing homogenous trifloaluminate ionic liquids appears to lie somewhere between χ_Al(OTf)3_ = 0.33 and 0.375, which corresponds to oligomeric structures [Al_2_(OTf)_10_]^4−^ and [Al_3_(OTf)_14_]^5−^, respectively (Figure 5). It is not suggested that these are the only structures present in at these compositions, but plausible examples of dominant anionic species, consistent with gathered evidence.

### Acceptor numbers

Whereas, the synthetic goal of preparing liquids with high Al(OTf)_3_ loadings was achieved, it was unsure whether the products would retain catalytic activity of Al(OTf)_3_. In order to assess Lewis acidity of the trifloaluminate ionic liquids, Gutmann acceptor number (AN) approach was adopted, which commonly used for Lewis acidic ionic liquids (Gutmann, 1978; Estager et al., 2010). AN values are measured using a weakly basic ^31^P NMR spectroscopic probe, triethylphosphine oxide (TEPO). Small amounts of TEPO were dissolved in each homogenous composition of both systems, and ^31^P NMR spectra were measured at three temperatures, 27, 57 and 87°C.

At the two lower temperatures, two ^31^P NMR signals were recorded across the compositional range, whereas one (probably averaged) signal was recorded at 87°C (SD, Figure S5). Following the approach adopted in our previous work (Hogg et al., 2017), AN values were calculated based on the more deshielded signal, corresponding to the strongest Lewis acid in the system.

Acceptor numbers ranged from AN = 64.9 to 69.3 for [C_8_mim][OTf]-Al(OTf)_3_, and from AN = 65.8 to 69.3 for [C_2_mim][OTf]-Al(OTf)_3_. These values were lower than those measured for Lewis acidic chloroaluminate ionic liquids (AN = 96), but comparable to mildly Lewis acidic chlorozincate ionic liquids (Estager et al., 2011). Furthermore, compared to chlorometallate systems, there was little variability across measured compositions, which suggests that in all samples species of similar acidity are present.

**Figure 6:**
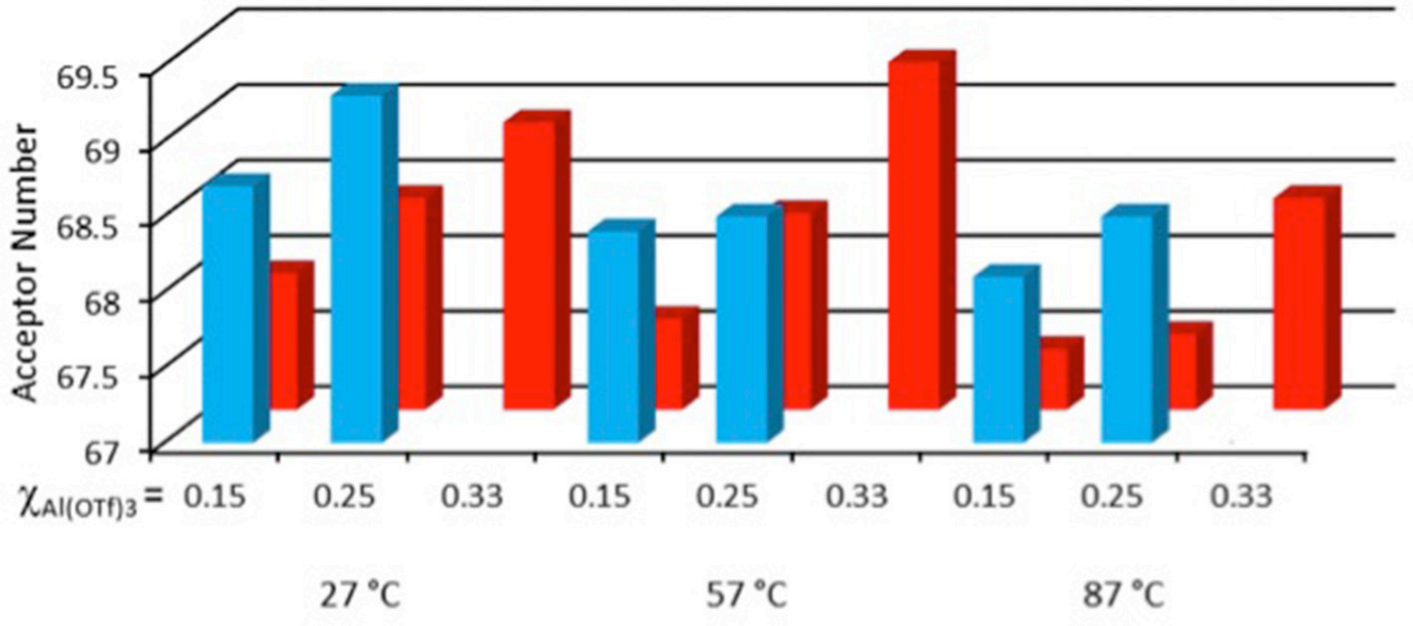
Acceptor numbers for [C_*n*_mim][OTf]-Al(OTf)_3_ (*n* = 2, blue; *n* = 8, red), measured for χ_Al(OTf)3_ = 0.15, 0.25 and 0.33, at three temperatures: 27, 57 and 87°C.

Analyzing the small variations between studied samples (Figure 6, actual AN values SD, Tables 1, 2), AN values increase with the increasing χ_Al(OTf)3_, and are on average slightly higher for [C_2_mim][OTf]-Al(OTf)_3_, compared to the analogous [C_8_mim][OTf]-Al(OTf)_3_ composition. Furthermore, AN values appear to decrease with temperature, which may relate to larger kinetic energy corresponding to lower propensity of the probe to bind to the aluminum center.

Aluminum surrounded exclusively by chloride ligands assumes maximum coordination of 4, and is 6-coordinate in the presence of *O*-donors. Lewis acidity in chloroaluminate ionic liquids arises from the coordinationally saturated [Al_2_Cl_7_]^−^ anion, where the bridging chloride bond must be broken to reveal latent Lewis acidic site. In analogy, it can be assumed that a bridging oxygen bond is severed in 6-coordinate a trifloaluminate anion, for the aluminum center to interact with a base. Difference in Lewis acidity is naturally explained by the presence of six *O*-donor ligands, which lower electrophilicity of the aluminum center more efficiently than four chloride ligands.

### Hydrolytic stability of trifloaluminate ionic liquids

Metal triflates are reported to be water tolerant Lewis acids; some of them can be even recovered from the aqueous phases after the reaction and recycled without loss of activity, e.g., La(OTf)_3_, Zn(OTf)_2_, Yb(OTf)_3_, and LiOTf (Kobayashi and Manabe, 2000; Kobayashi et al., 2002). Other metal triflates are known to slowly hydrolyse in water, releasing triflic acid, thus acting sometimes as a Brønsted acid–this includes Al(OTf)_3_, Sn(OTf)_2_, Sc(OTf)_3_, and In(OTf)_3_ (Baes and Mesmer, 1976; Noji et al., 2003; Markiton et al., 2018). As a rule of a thumb, metals at higher oxidation states are more prone to hydrolysis, with the exception of rare earth metal triflates, which are water-stable and act as Lewis acids in both aqueous and non-aqueous media (Kobayashi et al., 2002).

Trifloaluminate ionic liquids, in contrast to chloroaluminate analogs, do not react violently with water, but appear to be water-miscible in a wide range of proportions. Furthermore, trifloaluminate ionic liquids did not change their appearance upon overnight exposure to atmospheric moisture (air), neither showed marked change in their NMR spectra, whereas their chloroaluminate counterparts tend to undergo immediate hydrolysis evident by fuming and white crust forming on the interface of ionic liquid and air.

**Figure 7:**
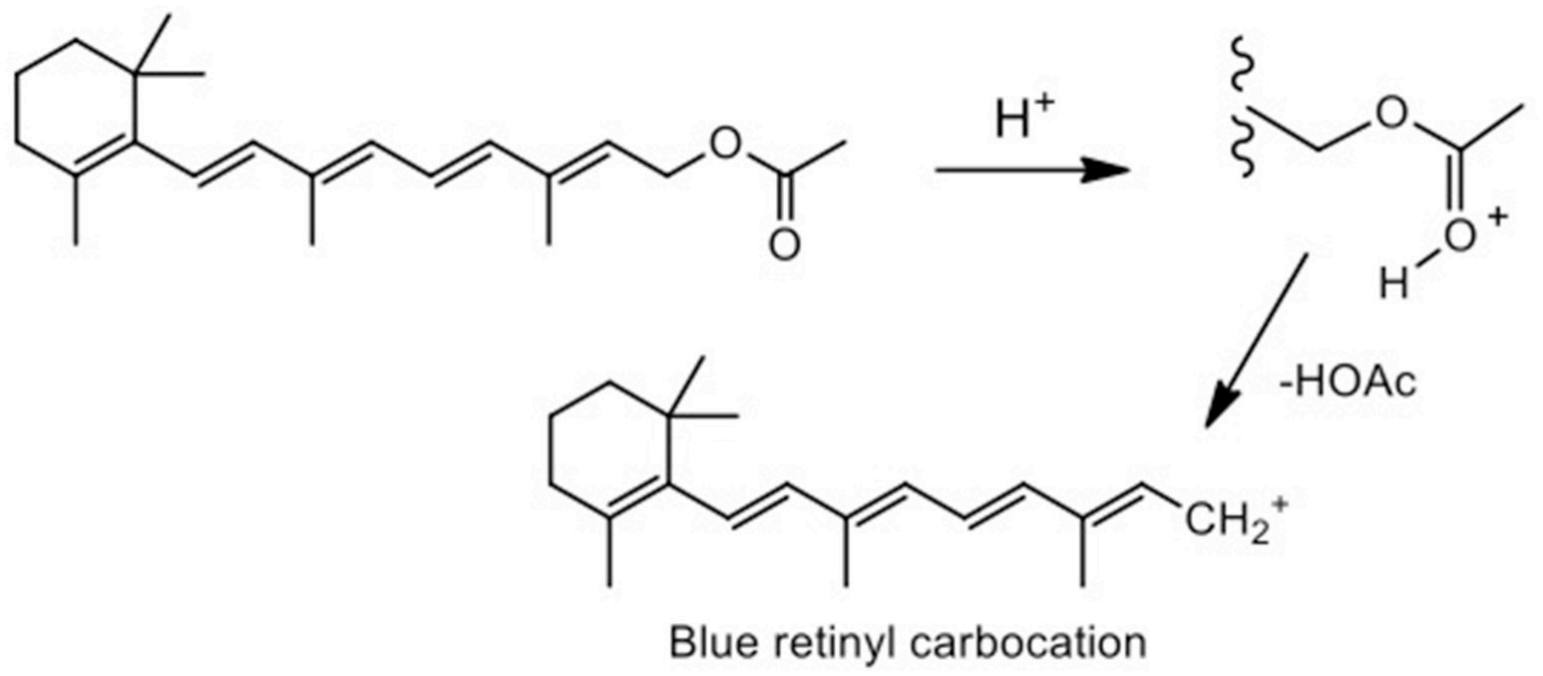
Formation of the retinyl carbocation.

In order to check for traces of hydrolysis undetectable by NMR spectroscopy, trifloaluminate ionic liquids (used as synthesized, without previous drying, exposed to atmospheric moisture) generation of retinyl cations from retinyl acetate in the presence of ionic liquid dissolved in nitrobenzene was used as the probe reaction. Nitrobenzene provided adequate solubility and did not generate protons by O–H bond polarization. Reaction of retinol with a Brønsted acid results in the formation of a blue carbocation, which can be detected and quantified by UV/Vis spectroscopy Figure 7, SD, Figure S7) (Williams and Lawton, 2010).

Three reactions involving [C_2_mim][OTf]-Al(OTf)_3_, χ_Al(OTf)3_ = 0.25, triflic acid and Al(OTf)_3_ (as benchmarks) all turned blue, indicating the formation of the retinyl cation, related to the presence of hydronium ion (Brønsted acidity). In contrast, reaction with lanthanum triflate did not cause color change, which is associated with the absence of hydrolysis products. In conclusion, although trifloaluminate ionic liquids do not violently react with water, they may undergo very slow hydrolysis in the presence of atmospheric moisture.

### Synthesis of a model chromane, catalyzed by metal triflates

**Figure 8:**
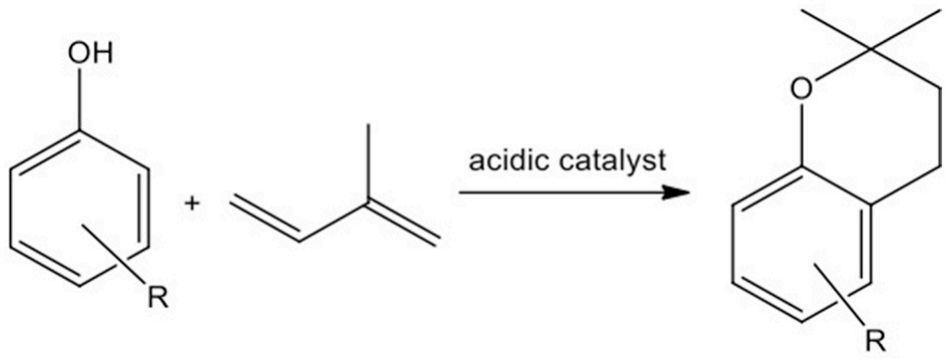
Chromane synthesis from 2-methyl-1,3-diene and a phenol derivative.

An important reaction typically catalyzed by metal triflates is the synthesis of chromans *via* the reaction of dienes with phenolic compounds (Figure 8; Youn and Eom, 2006; Bonrath et al., 2007; Adrio and Hii, 2008; Vece et al., 2010; Dang et al., 2011). The two-step reaction involves alkylation of a phenolic compound, followed by etherification reaction to form benzofuran or benzopyran. 2,2-Dimethylbenzopyran is found in the natural products, such as vitamin E (Schneider, 2005) and flavonoids (Middleton et al., 2000); some of the latter compounds are biologically active (Brown et al., 1990), for example as HIV protease inhibitors (Kashman et al., 1992).

Reaction carried out in the presence of conventional catalysts, both Lewis and Brønsted acids (Amberlyst® 15, H_3_PO_4_, AlCl_3_) (Ahluwalia et al., 1982; Kalena et al., 1997; Bonrath et al., 2007) requires high temperatures and high catalyst loadings. Selectivity is also a challenge, with side-products forming *via C*-alkylation of phenol by diene, toward substituted alkylated phenols, as well as alkylated chromans.

Amongst the most promising catalysts are metal triflates; for example, AgOTf (5 mol% in dichloroethane, at ambient temperature) promoted the formation of a variety of dihydrobenzopyran and dihydrobenzofuran ring systems; for example, addition of isoprene to 4-methoxyphenol gave 61% of yield of benzopyran (Youn and Eom, 2006). Yet, although various Lewis acidic metal triflates have been used as catalysts for the synthesis of chromanes (Youn and Eom, 2006; Bonrath et al., 2007; Adrio and Hii, 2008; Vece et al., 2010; Dang et al., 2011), the literature lacks comparative studies of the activity of different metal triflates, which could guide the selection of best triflometallate ionic liquids.

**Figure 9:**
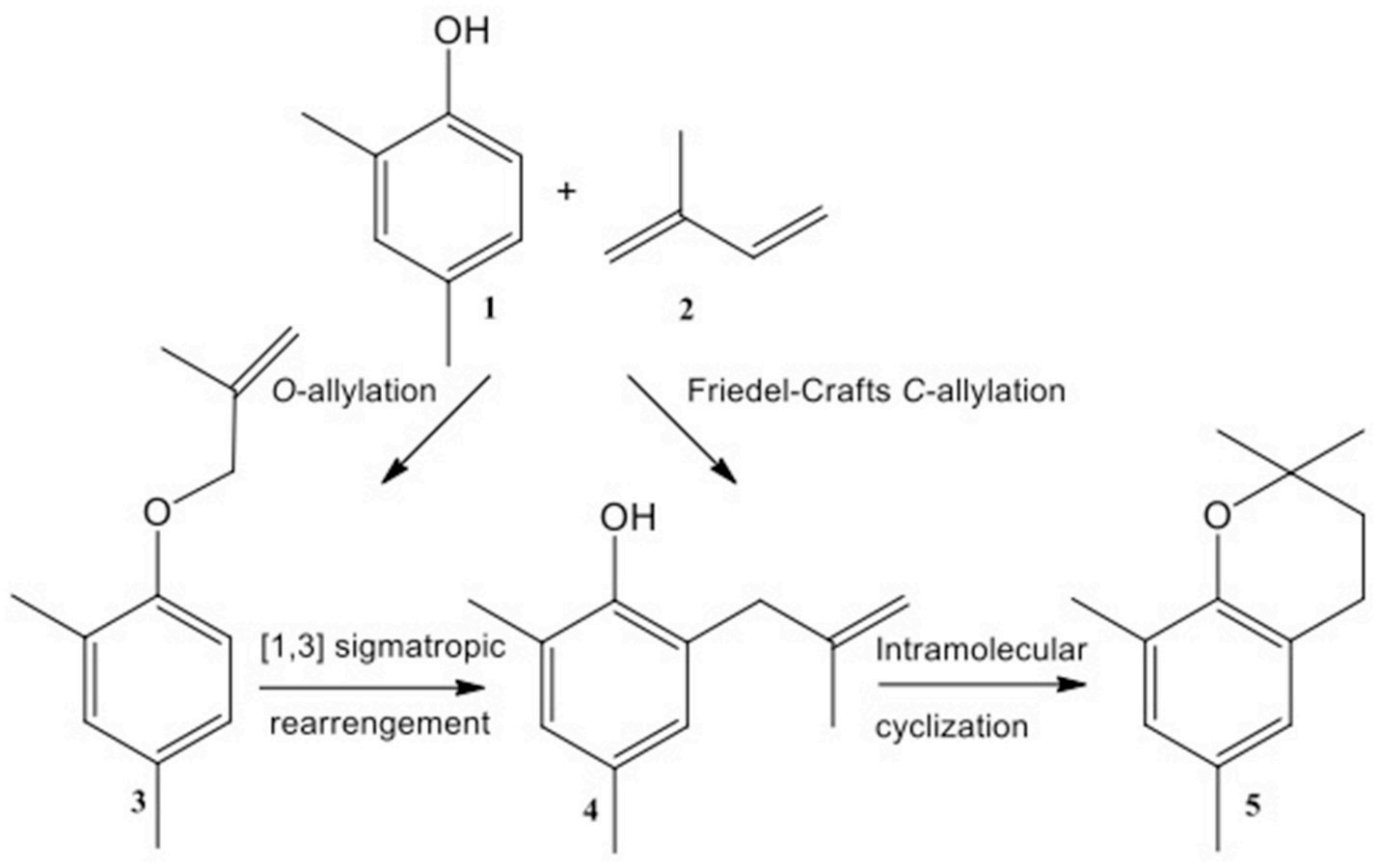
The model reaction of 2,4-dimethylphenol with 2-methyl-1,3-butadiene.

In the preliminary screening, a wide range of metal triflates: Al(III), Sn(II), Sc(III), In(III), Li(I), Ag(I), Ga(III), Zn(II), La(III), Bi(III), Yb(III), Y(III), Tl(III) and Er(III), were tested as Lewis acidic catalysts in the synthesis of the model compound: 2,2-dimethyl-2,4-dimethylchromane, obtained through the reaction of 2,4-dimethylphenol with 2-methyl-1,3-butadiene (isoprene). There are two pathways leading to the chromane formation (Figure 9; Vece et al., 2010). The first route concerns Friedel–Crafts *C*-allylation to **4** followed by intramolecular cyclisation to chromane, **5**. The second sequence requires the formation of allyl-aryl ether, **3**, followed by a [3,3]-sigmatropic rearrangement to **4**, and further intramolecular cyclisation leading to **5**. Products **3** and **4** are the intermediates of this reaction.

The model reaction was carried out at 35°C, solventless, using 1:2 molar ratio of isoprene:2,4-dimethylphenol. In all cases, the addition of 2 mol% of metal triflate gave a suspension of catalyst in the liquid reaction mixture, rather than a homogenous solution. Conversion of 2,4-dimethylphenol (α) was measured after 90 min of the reaction, using GC (Table 3).

**Table 3:** The synthesis of model chromane in the presence of various metal triflates.

**Entry**	**Metal triflate**	**α, %**	**Selectivity, %**
1	Al(OTf)_3_ (90 min)	70	80
	(120 min)	78	80
	(150 min)	82	80
	(180 min)	82	80
2	Sn(OTf)_2_	100	81
3	Sc(OTf)_3_	100	91
4	Bi(OTf)_3_	100	92
5	Ga(OTf)_3_	100	89
6	In(OTf)_3_	97	81
7	Yb(OTf)_3_	100	66
8	Ag(OTf)	30	42
9	Li(OTf)	10	14
10	Y(OTf)_3_	7	14
11	Er(OTf)_3_	6	100
12	Zn(OTf)_2_, La(OTf)_3_, Tl(OTf)_3_	0	0

*Reaction conditions: 35°C, 2,4-dimethylphenol (4 mmol), isoprene (2 mmol), catalyst loading 2 mol % per isoprene, 1,500 rpm, reaction time 90 min*.

Sn(OTf)_2_, Sc(OTf)_3_, Bi(OTf)_3_, Ga(OTf)_3_, and In(OTf)_3_ exhibited the highest activity, achieving full conversion of 2,4-dimethylphenol, combined with high selectivities to chromane (81–92%) after 90 min. Rare-earth metals were found inactive under the reaction conditions, except for Yb(OTf)_3_, which offered poor selectivity. Al(OTf)_3_ gave intermediate results, with conversion of 82% (at 80% selectivity) achieved after 150 min. Two by-product were detected (GC-MS analysis, ESI) across all post-reaction mixtures: 2,4-dimethyl-1-[(3-methyl-3-buten-1-yl)oxy]benzene (**3**) and 2,4-dimethyl-6-(3-methyl-3-buten-1-yl)phenol (**4**); both of them are intermediates leading to the target chromane (Figure 9). Therefore, the challenge appears to lie in shifting the equilibrium toward the product formation, rather than avoiding unwanted by-products.

As already mentioned, the focus on triflometallate ionic liquids was dictated by the drive to find both chemically active and economically attractive system. Seeing that the most active metal triflates were also among the most expensive ones, in this work we set to explore whether the ionic liquid pathway may offer an enhancement of catalytic activity of the inexpensive Al(OTf)_3_, before venturing into more expensive metal triflates.

### Synthesis of a model chromane, catalyzed by trifloaluminate ionic liquids

AN values measured for all triflometallate ionic liquids were relatively similar, with small variations resulting from the length of alkyl chain on the cation, temperature of measurement or even on the χ_Al(OTf)3_ value. Considering viscosity rising dramatically with increasing χ_Al(OTf)3_, it was decided to select systems with lower Al(OTf)_3_ loading: χ_Al(OTf)3_ = 0.15 and 0.25, which bore promise of better mass transport in a biphasic liquid mixture, due to lower viscosity. By the same token, systems based on [C_2_mim]^+^ cation were selected over [C_8_mim]^+^ cation.

#### Reactants ratio

**Figure 10:**
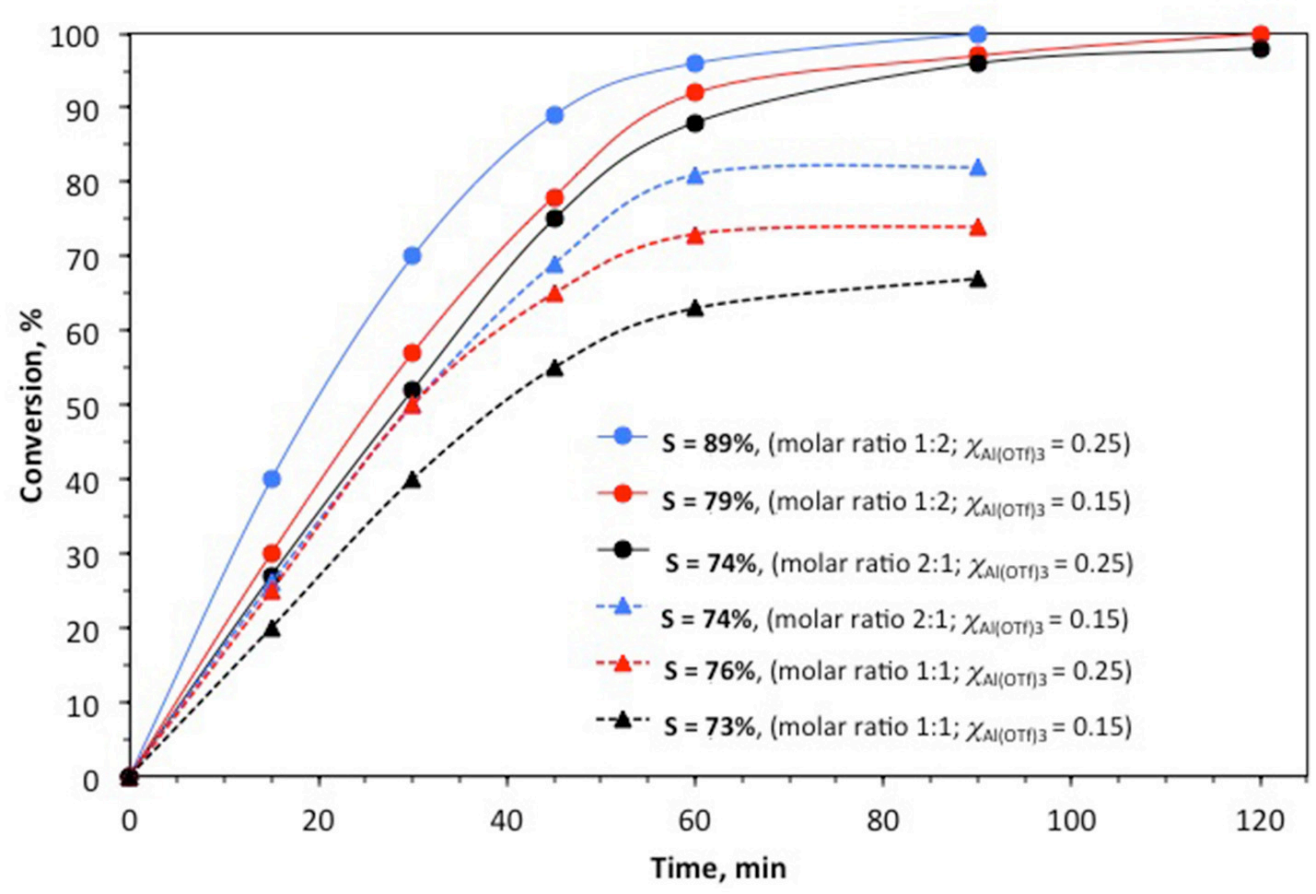
The influence of molar ratio of isoprene to 2,4-dimethylphenol (in parentheses) on the conversion of 2,4-dimethylphenol and selectivity to chromane. *Reaction conditions:* 35°C, catalyst loading 2 mol % per isoprene, 1,500 rpm. Catalyst: [C_2_mim][OTf]-Al(OTf)_3_.

In the first set of experiments, trifloaluminate ionic liquids were used as homogenous catalysts in the synthesis of chromane under solventless conditions, at 35°C, and molar ratio of isoprene to 2,4-dimethylphenol was varied between 1:2 and 2:1 (Figure 10). Best results were recorded for 2-fold molar excess of 2,4-dimethylphenol to isoprene, which allowed for reaching full conversion of 1 mol of 2,4-dimethylphenol per 1 mol of isoprene and high selectivities: 89% when using [C_2_mim][OTf]-Al(OTf)_3_, χ_Al(OTf)3_ = 0.25, and 79% when using [C_2_mim][OTf]-Al(OTf)_3_, χ_Al(OTf)3_ = 0.15. Using the catalyst with lower aluminum loading resulted in longer reaction time required to reach full conversion (120 vs. 90 min), which can be justified in lower content of Lewis acidic aluminum centers per mole of the χ_Al(OTf)3_ = 0.15 ionic liquid (calculated as mole of cation).

#### Reaction conditions

The influence of reaction temperature and catalyst loading was studied, keeping the reactants ratio at two-fold molar excess of 2,4-dimethylphenol (Table 4). Selectivity to the main product (**5**) and the two by-products (**3, 4**) was reported. For the sake of comparison, reaction was carried out in the presence of HOTf and Al(OTf)_3_, since both Lewis and Brønsted acid sites may be involved in catalysis (Table 4, entries 1-4). Ionic liquids with both cations: [C_2_mim]^+^ and [C_8_mim]^+^ were screened, at two compositions: χ_Al(OTf)3_ = 0.15 and 0.25 (Table 4, entries 5–12). In general, increase of selectivity with reaction time was observed, as intermediates **3** and **4** were transformed to **5** in the course of reaction.

**Table 4:** The influence of the temperature and amount of various catalysts on the conversion of 2,4-dimethylphenol.

**Entry**	**Catalyst**	**T°C**	**Catalyst loading, mol% per isoprene**	**Time, min**	**α,%**	**Selectivity,%**
1	TfOH	35	0.2	180	81	82(**5**), 9 (**3**), 9 (**4**)
2	Al(OTf)_3_	20	2.0	60	10	23
3		35	2.0	150	82	80 (**5**), 10 (**3**), 10 (**4**)
4		35	0.2	240	30	48
5	[C_2_mim][OTf]-Al(OTf)_3_, χ_Al(OTf)3_ = 0.15	35	2.0	120	99	79 (**5**), 8 (**3**), 13 (**4**)
6	[C_2_mim][OTf]-Al(OTf)_3_, χ_Al(OTf)3_ = 0.25	20	2.0	120	99	78 (**5**), 10 (**3**), 12 (**4**)
7		35	2.0	60	94	89 (**5**), 4 (**3**), 7 (**4**)
8		35	1.0	90	96	81 (**5**), 8 (**3**), 11 (**4**)
9		35	0.2	180	95	84 (**5**), 6 (**3**), 10 (**4**)
10		35	0.15	180	61	73 (**5**), 10 (**3**), 17 (**4**)
11	[C_8_mim][OTf]-Al(OTf)_3_, χ_Al(OTf)3_ = 0.15	35	2.0	120	97	81 (**5**), 8 (**3**), 11 (**4**)
12	[C_8_mim][OTf]-Al(OTf)_3_, χ_Al(OTf)3_ = 0.25	35	2.0	60	95	87 (**5**), 4 (**3**), 9 (**4**)
13	MWCNT-[C_2_mim][OTf]-Al(OTf)_3_, χ_Al(OTf)3_ = 0.25	35	0.2	90	99	84 (**5**), 10 (**3**), 6 (**4**)
14	MWCNT-[C_2_mim][OTf]-Al(OTf)_3_, χ_Al(OTf)3_ = 0.25	35	0.2*^a^*	120	66	80 (**5**), 13 (**3**), 7 (**4**)
15	MWCNT-[C_2_mim][OTf]-Al(OTf)_3_, χ_Al(OTf)3_ = 0.25	35	0.15	90	76	78(**5**), 9 (**3**), 13 (**4**)

a *recycled catalyst. Reaction conditions: 35°C, 2,4-dimethylphenol (4 mmol), isoprene (2 mmol), 1,500 rpm*.

Comparing activity of Al(OTf)_3_ and [C_2_mim][OTf]-Al(OTf)_3_, χ_Al(OTf)3_ = 0.25, lower activity of the former is particularly visible under less favorable reaction conditions. At low reaction temperature (20°C) the ionic liquids retained high activity, in contrast to Al(OTf)_3_ (Table 4, entries 2 and 6). Furthermore, at low catalyst loading (0.2 mol%) Al(OTf)_3_ gave 30% conversion after 240 min, whereas the ionic liquid delivered 95% conversion after 180 min (Table 4, entries 4 and 9).

Comparing catalytic activity of different IL catalysts, the cation had no measurable influence on the catalytic performance. As expected, ILs with lower Al(OTf)_3_ loading were less active under the same reaction conditions. Below 0.2 mol% loading, it was impossible to reach full conversion using [C_2_mim][OTf]-Al(OTf)_3_, χ_Al(OTf)3_ = 0.25 under standard reaction conditions (reaction has stopped at 75% conversion).

#### Moisture sensitivity

Since no evidence for hydrolysis was found, either visually or through the NMR spectroscopy, chromane syntheses were carried out without protective atmosphere or drying precautions. However, following the retinyl cation test indicating the presence of acidic protons, it was decided to repeat two experiments with trifloaluminate ionic liquids (Table 4, entries 7 and 9) in a glovebox, under strictly anhydrous conditions. Nevertheless, no differences in conversion or selectivity were detected compared to unprotected experiments, indicating that protic impurities must have been generated at trace levels only, and did not interfere with catalytic performance of ionic liquids.

#### Recycling

It was attempted to recycle the [C_2_mim][OTf]-Al(OTf)_3_, χ_Al(OTf)3_ = 0.25 catalyst, from reaction carried out at 0.2 mol% loading. After completion, *n*-hexane was added to the reaction mixture and two phases formed. The lower (ionic liquid) phase was separated and re-used. Unfortunately, activity of the recycled catalyst decreased (Table 4, entry 14), yielding 66% conversion of 2,4-dimethylphenol (and selectivity of 80%) after 120 min.

Seeking strategies for a more efficient recycle of trifloaluminate ionic liquids, it was attempted to use it as supported ionic liquid phase (SILP), with multi-walled carbon nanotubes (MWCNTs) used as the support.

### Synthesis of model chromane, catalyzed by trifloaluminate ionic liquids immobilized on carbon nanotubes

Carbon nanotubes (CNTs) have been described as versatile supports for immobilization of catalysts due to their small size, large surface area, as well as mechanical and thermal stability. Supporting catalysts on CNTs has had profound effect on both catalytic activity and selectivity, with major enhancements reported in the literature (Eder, 2010). The economy of CNT-based processes is increasingly favorable, with prices of industrial-grade MWCNTs as low as 100$ per 1 kg (Nanocyl^TM^ NC7000 MWCNTs). Finally, in CNTs prepared *via* catalytic chemical vapor deposition (c-CVD), their integral feature is the presence of core-encapsulated ferromagnetic iron-based nanoparticles, which are typically irremovable under lower technological regimes. This characteristic allows for an easy removal of the catalysts from the post-reaction mixtures by means of a magnetic field, which is a significant advantage in light of the processing economy.

The superactive catalysts based on carbon nanomaterials as supports have been already demonstrated in our earlier studies (Markiton et al., 2017, 2018). Extremely high activity of Sn(OTf)_2_ immobilized on MWCNTs in promoting the Baeyer-Villiger oxidation was related to spherical nanosize Sn(OTf)_2_ particles (*ca*. 2 nm) dispersed along the outer nanotube walls.^13^ In chemo-enzymatic Baeyer-Villiger reaction, the performance of lipase was significantly improved by immobilization on MWCNTs (Markiton et al., 2017).

Encouraged by the past results, we decided to support trifloaluminate ionic liquids on MWCNTs, in hope to develop a high-performance and recyclable SILP catalyst. To the best of our knowledge, it is the first attempt to use solid-immobilized metal triflate for chromans synthesis.

#### Comparison of homogenous and SILP systems

The best-performing ionic liquid, [C_2_mim][OTf]-Al(OTf)_3_, χ_Al(OTf)3_ = 0.25, was immobilized on the CheapTubes™ MWCNTs, at a mass ratio of MWCNTs to ionic liquid of 1:2. Suspension of the ionic liquid and MWCNTs was suspended in *n*-hexane by ultrasonication, and then filtered. Subsequent washing with *n*-hexane and solvent removal under reduced pressure yielded the heterogenised catalyst, MWCNT-[C_2_mim][OTf]-Al(OTf)_3_, χ_Al(OTf)3_ = 0.25. The quantity of the active phase (ionic liquid) was estimated by thermogravimetric analysis (TGA, SI, Figures S8–S12), and the SILP catalyst was characterized in comparison to neat MWCNTs. As compared in Table S5, both pore volumes (V_p_) and overall surface area (SBET) decreased in SILP compared to neat MWCNTs, as the result of interstitial channels–forming both pores and available surface–occupied by the adsorbed ionic liquid (33 wt%).

The trifloaluminate ionic liquid in the SILP form, MWCNTs-[C_2_mim][OTf]-Al(OTf)_3_, χ_Al(OTf)3_ = 0.25, was tested as the catalyst for chromane synthesis under solventless conditions, at low catalyst loading of 0.2 mol%, (Table 4, entry 13). It was found that immobilization of the trifloaluminate ionic liquid increased its catalytic activity, with the reaction time required for full conversion shortened to 90 min, compared to 180 min needed in analogous homogenous reaction (Table 4, entry 9).

#### Optimisation of the IL loading

The original procedure used to prepare the SILP catalyst was modified to combine MWCNTs and the ionic liquid at mass ratios of 4:1 and 1:1. The sample prepared at 4:1 ratio contained only 16 wt% of ionic liquid, and required 0.2 mol% loading to work as a catalyst, which also caused stirring problems due to large volume of suspended MWCNTs in the synthesis of chromane. In contrast, the use of catalyst prepared at 1:1 ratio, contained 30 wt% of the ionic liquid (nearly the same as sample prepared with two-fold excess of ionic liquid) let efficient mixing of reaction system. In conclusion, it was found sufficient to use equal mass of the ionic liquid and MWCNTs to prepare “fully loaded” SILP catalyst.

#### Leaching and recycling

Leaching was tested by repeating the benchmark reaction with the [C_2_mim][OTf]-Al(OTf)_3_, χ_Al(OTf)3_ = 0.25 catalyst (Table 4, entry 5), but filtering the catalyst off after 15 min (conversion 37%, selectivity 61%), and continuing the reaction in absence of the catalyst. After 30 min (15 min after the filtration) the reaction progressed slightly further (conversion 44%, selectivity 63%), but further progress was significantly halted, with conversion 51% and selectivity 65% reported after 90 min and no further change after 150 min. For comparison, the benchmark experiment (Table 3, entry 5) gave full conversion and 84% selectivity after 90 min. In conclusion, the slow progress of the reaction after SILP catalyst removal could be attributed to some amount of leaching.

**Figure 11:**
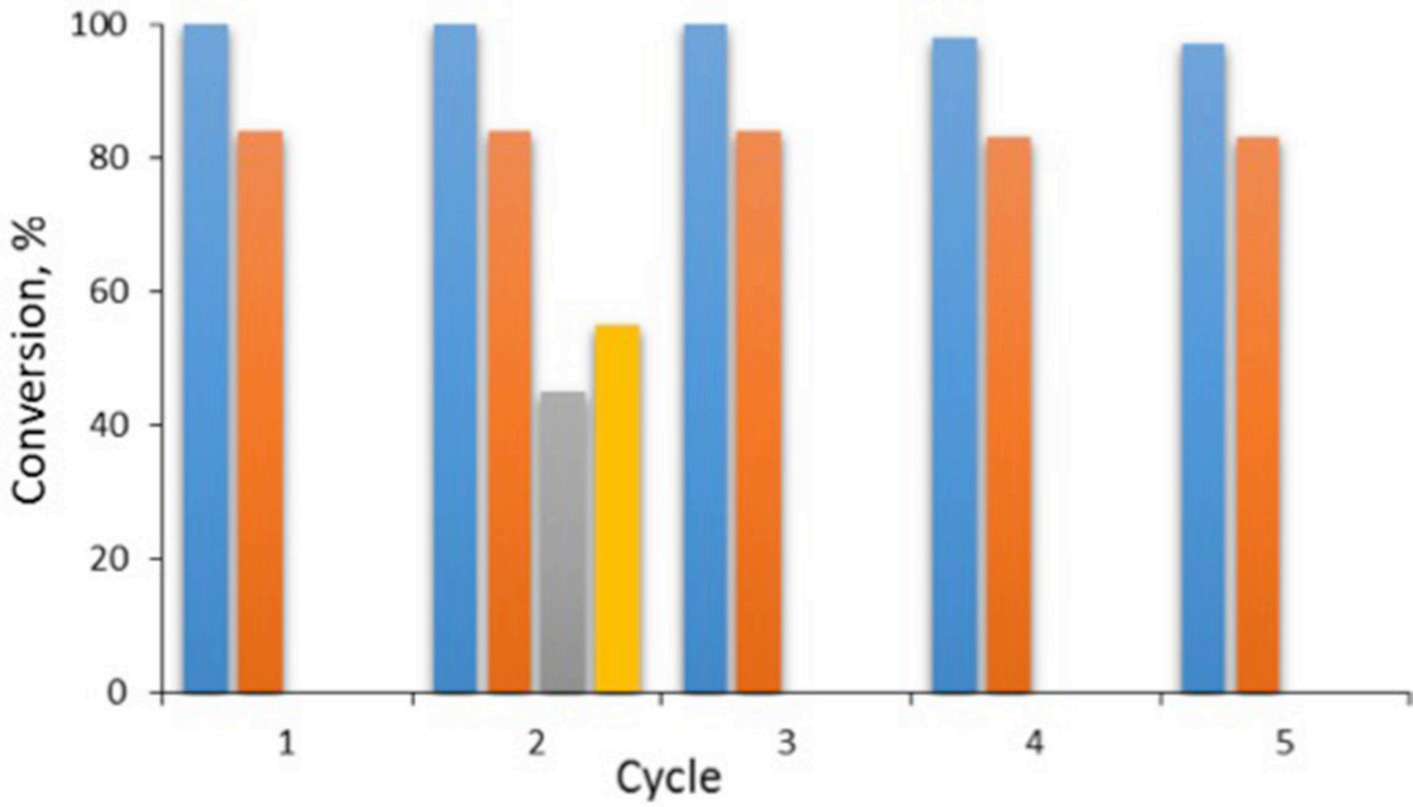
The recycle of catalyst MWCNT-[C_2_mim][OTf]-Al(OTf)_3_, χ_Al(OTf)3_ = 0.25 after regeneration; blue bars: conversion; orange bars: selectivity; gray bar: conversion and yellow bar: selectivity for the reaction carried out with the recycled catalyst without its regeneration. *Reaction conditions:* 35°C, 2,4-dimethylphenol (4 mmol), isoprene (2 mmol), catalyst loading 0.2 mol% per isoprene, 1,500 rpm, reaction time 90 min.

Indeed, TGA analysis of the filtered-off catalyst sample revealed that *ca*. 27 wt% of the initial amount of ionic liquid was removed from the MWCNT surface. In agreement with this assessment, recycled catalyst gave lower performance than the fresh sample (conversion 45%, selectivity 56%), as shown in Figure 11.

Fortunately, it was found that the leached ionic liquid could be recovered from the post-reaction mixture and re-immobilized on the MWCNTs surface, which restored full catalytic activity. After each reaction cycle, the SILP catalyst was filtered off and *n*-hexane was added to the filtrate. Next, the lower phase (containing ionic liquid) was isolated and immobilized on the surface of the SILP sample that has been isolated by filtration. Such re-immobilized system contained 33 wt% of the ionic liquid loading (by TGA), and retained high catalytic activity and selectivity over four recycles (Figure 11).

### Comparison to the literature

Comparing literature reports on various tandem additions/cyclizations of phenols with dienes, it is evident that using trifloaluminate ionic liquids offers certain improvements, either in homogenous or SILP form.

For comparison, AgOTf was used at 5 mol% loading (RT, 1,2-dichroetane as a solvent), and gave 54–96% yields after 24 h (S. Youn and Eom, 2006). It was possible to reduce AgOTf loading to 1 mol%, but the addition of 4 mol% of ^*t*^BuCl was required, which gave up to 86% conversion to dihydrobenzopyrans in 20 min. (Dang et al., 2011). Nevertheless, in both cases higher loadings of a more expensive catalyst are required, in addition to cancerogenic chlorinated solvents/co-catalysts.

In other examples, In(OTf)_3_ was used at 1 mol% (RT, 2 h, average yield 80%; Vece et al., 2010), and Bi(OTf)_3_ was used at 5 mol% (RT, 2 h or RT, 10-16 h, depending on reactants, gave 80% yield; Ollevier and Mwene-Mbeja, 2006). The lowest catalyst loadings were achieved with Cu(OTf)_2_ promoted with PPh_3_, which worked at 0.5 mol% loading (RT, 18 h; Adrio and Hii, 2008).

## Conclusions

In this contribution, we report the first example of triflometallate ionic liquids. Trifloaluminate systems, in contrast to the chloroaluminate counterparts, contain hexacoordinate aluminum in multiply-charged, oligonuclear anionic complexes, featuring a variety of triflate bridging modes. As a consequence of this speciation, trifloaluminate ionic liquids are viscous, and it is only practical to use them at low Al(OTf)_3_ loadings (up to χ_Al(OTf)3_ = 0.25). Their acidity is lower than that of chloroaluminate ionic liquids, and similar to chlorozincate ionic liquids.

Newly reported trifloaluminate systems have been found efficient catalysts in the chromane syntheses in solvent-free, one-pot process. In contrast to literature processes, the catalyst loadings were found to be truly catalytic (0.2 mol%) and the reaction times required for the full conversion were drastically shortened. The procedure is entirely chloride-free, and eliminates the requirement for chlorinated solvent/promoter, reported in some earlier works.

Further improvements were achieved by supporting trifloaluminate ionic liquids on multi-walled carbon nanotubes. In agreement with earlier reports, the activity of the catalyst was enhanced by the support, and recycling was facilitated by enabling simple filtration. Although some ionic liquid leached into the reaction mixture, it was possible to recover and re-support it, which resulted in a recyclable catalytic system.

## Author contributions

PL and NB synthesis of chromane; AlC and LB synthesis and speciation of ionic liquids; SJ thermal analysis; SB preparation and analysis of supported ionic liquids; PN and MS-K supervision of PL, AlC and LB, contribution to manuscript writing; AnC supervision of PL and NB, contribution to manuscript writing.

### Conflict of interest statement

The authors declare that the research was conducted in the absence of any commercial or financial relationships that could be construed as a potential conflict of interest.
