# Supplementary material for: Water-Tolerant Trifloaluminate Ionic Liquids: New and Unique Lewis Acidic Catalysts for the Synthesis of Chromane
Source: Front Chem. 2018 Nov 12;6:535. doi: 10.3389/fchem.2018.00535 (PMC6240586; doi:10.3389/fchem.2018.00535)
Supplement: Supplementary file 1 [file Table_1.DOCX]

Supplementary Material

**Water-Tolerant Trifloaluminate Ionic Liquids: New and Unique Lewis Acidic Catalysts for the Synthesis of Chromane**

**Piotr Latos^1^, Alice Culkin^2^, Natalia Barteczko^1^, Sławomir Boncel^3^, Sebastian Jurczyk^4^, Lucy C. Brown^2^, Peter Nockemann^2^, Anna Chrobok^1^*, Małgorzata Swadźba-Kwaśny^2^***

^1^ Department of Organic Chemical Technology and Petrochemistry, Silesian University of Technology, Gliwice, Poland,

^2^ The QUILL Research Centre, School of Chemistry and Chemical Engineering, Queen's University Belfast, Belfast, UK,

^3^ Department of Organic Chemistry, Bioorganic Chemistry and Biotechnology, Silesian University of Technology, Gliwice, Poland,

^4^ Institute for Engineering of Polymer Materials and Dyes, Toruń, Poland

*** Correspondence:**Anna Chrobok

anna.chrobok@polsl.pl

Małgorzata Swadźba-Kwaśny

m.swadzba-kwasny@qub.ac.uk

**1. Acceptor numbers**

**Table S1. ^31^P-NMR chemical shifts of various compositions of** [C_8_mim][OTf]-Al(OTf)_3_ **and the resulting acceptor numbers after adding different quantities (mol%) of TEPO.**

| **Χ_Al(OTf)3_** | **Temperature (°C)** | **^31^P Chemical shift (ppm) 1% TEPO** | **^31^P Chemical shift (ppm) 2% TEPO** | **^31^P Chemical shift (ppm) 3% TEPO** | **Acceptor Number** |
| --- | --- | --- | --- | --- | --- |
| **0.15** | **27** | 71.912 | 71.928 | 71.916 | **67.9** |
|  |  | 70.830 | 70.874 | 70.924 | **65.3** |
|  | **57** | 71.832 | 71.839 | 71.936 | **67.6** |
|  |  | 70.671 | 70.778 | 70.804 | **64.9** |
|  | **87** | 71.683 | 71.610 | 71.662 | **67.4** |
|  |  | 70.993 | 70.777 | 70.957 | **65.7** |
| **0.25** | **27** | 72.093 | 72.119 | 72.083 | **68.4** |
|  |  | 70.802 | 71.038 | 70.983 | **65.2** |
|  | **57** | 72.046 | 72.027 | 71.972 | **68.3** |
|  |  | 70.936 | 70.820 | 70.933 | **65.5** |
|  | **87** | 71.706 | 71.790 | 71.690 | **67.5** |
| **0.33** | **27** | 72.216 | 72.230 | 72.075 | **68.9** |
|  |  | 71.185 | 71.089 | 71.001 | **66.4** |
|  | **57** | 72.314 | 72.267 | 72.043 | **69.3** |
|  |  | 71.471 | 71.299 | 70.918 | **67.6** |
|  | **87** | 72.019 | 72.023 | 71.879 | **68.4** |

**Table S2**. ^31^P-NMR chemical shifts of various compositions of [C_2_mim][OTf]-Al(OTf)_3_ and the resulting acceptor numbers after adding different mass % of TEPO.

| **Χ_Al(OTf)3_** | **Temperature (°C)** | **^31^P Chemical shift (ppm) 1% TEPO** | **^31^P Chemical shift (ppm) 2% TEPO** | **^31^P Chemical shift (ppm) 3% TEPO** | **Acceptor Number** |
| --- | --- | --- | --- | --- | --- |
| **0.15** | **27** | 72.264 | 72.275 | 72.276 | **68.7** |
|  |  | 71.275 | 71.259 | 71.322 | **66.3** |
|  | **57** | 72.274 | 72.279 | 72.286 | **68.8** |
|  |  | 71.081 | 71.246 | 71.260 | **65.8** |
|  | **87** | 72.036 | 72.023 | 72.099 | **68.1** |
| **0.25** | **27** | 72.455 | 72.410 | 72.374 | **69.3** |
|  |  | 71.332 | 71.359 | 71.321 | **66.6** |
|  | **57** | 72.237 | 72.432 | 72.332 | **68.7** |
|  |  | 71.524 | 71.270 | 71.368 | **67.1** |
|  | **87** | 72.120 | 72.220 | 72.129 | **68.5** |

**2. Synthesis of ionic liquids**

**Synthesis of [C_2_mim][OTf]-Al(OTf)_3_**

1 g samples were prepared by weighing the appropriate masses of 1-methyl-3-ethylimidazolium triflate and aluminium triflate into a vial in the glove box, with the exception of χ_Al(OTf)3_ = 0.5, where a 2 g sample was prepared. The triflometallates were stirred at 85 °C to homogenise for 2 h.

**Table S3.**

| **χ_Al(OTf)3_** | **[C_2_mim][OTf], mass, g** | **Al(OTf)_3,_ mass, g** | **[C_2_mim][OTf], mol** | **Al(OTf)_3_ , mol** |
| --- | --- | --- | --- | --- |
| 0.15 | 0.7564 | 0.2433 | 2.91 x 10^-3^ | 5.13 x 10^-4^ |
| 0.20 | 0.6885 | 0.3133 | 2.65 x 10^-3^ | 6.61 x 10^-4^ |
| 0.25 | 0.6226 | 0.3781 | 2.39 x 10^-3^ | 7.97 x 10^-4^ |
| 0.33 | 0.5263 | 0.4755 | 2.02 x 10^-3^ | 1.00 x 10^-3^ |
| 0.40 | 0.4528 | 0.5483 | 1.74 x 10^-3^ | 1.16 x 10^-3^ |
| 0.50 | 0.7116 | 1.2909 | 2.73 x 10^-3^ | 2.72 x 10^-3^ |

**Synthesis of [C_8_mim][OTf]-Al(OTf)_3_**

1 g samples of each composition were prepared in the same fashion as above.

**Table S4.**

| **χ_Al(OTf)3_** | **[C_8_mim][OTf], mass, g** | **Al(OTf)_3_, mass, g** | **[C_8_mim][OTf], mol** | **Al(OTf)_3_, mol** |
| --- | --- | --- | --- | --- |
| 0.15 | 0.8189 | 0.1997 | 2.38 x 10^-3^ | 4.21 x 10^-4^ |
| 0.25 | 0.6859 | 0.3143 | 1.99 x 10^-3^ | 6.63 x 10^-4^ |
| 0.33 | 0.5978 | 0.4038 | 1.73 x 10^-3^ | 8.52 x 10^-4^ |
| 0.4 | 0.5306 | 0.4786 | 1.54 x 10^-3^ | 1.00 x 10^-3^ |
| 0.5 | 0.4242 | 0.5791 | 1.23 x 10^-3^ | 1.22 x 10^-3^ |


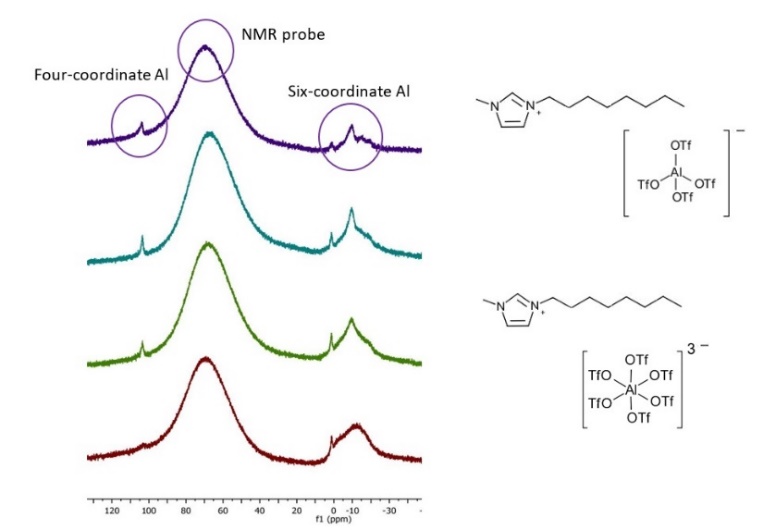


**Figure S1.** ^27^Al-NMR spectra of [C_8_mim][OTf]-Al(OTf)_3_, χ_Al(OTf)3_ = 0.15 at 27, 47, 57, 67 and 87 °C (neat using a DMSO capillary).


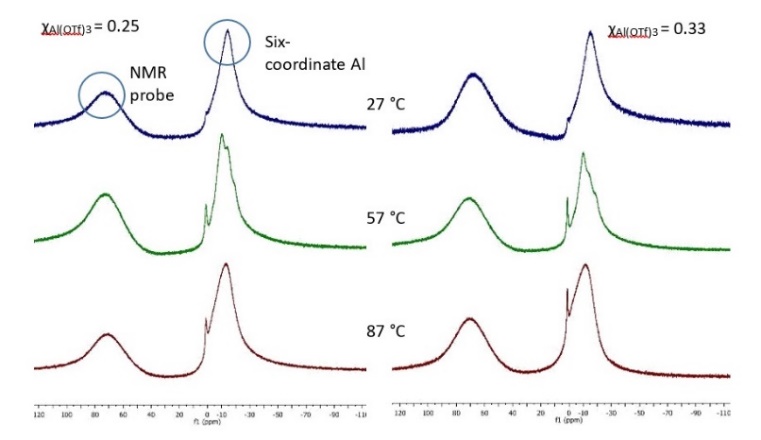


**Figure S2.** ^27^Al-NMR spectra of [C_8_mim][OTf]-Al(OTf)_3_, χ_Al(OTf)3_ = 0.25 and 0.33, at 27, 57 and 87 °C (neat using a DMSO capillary).


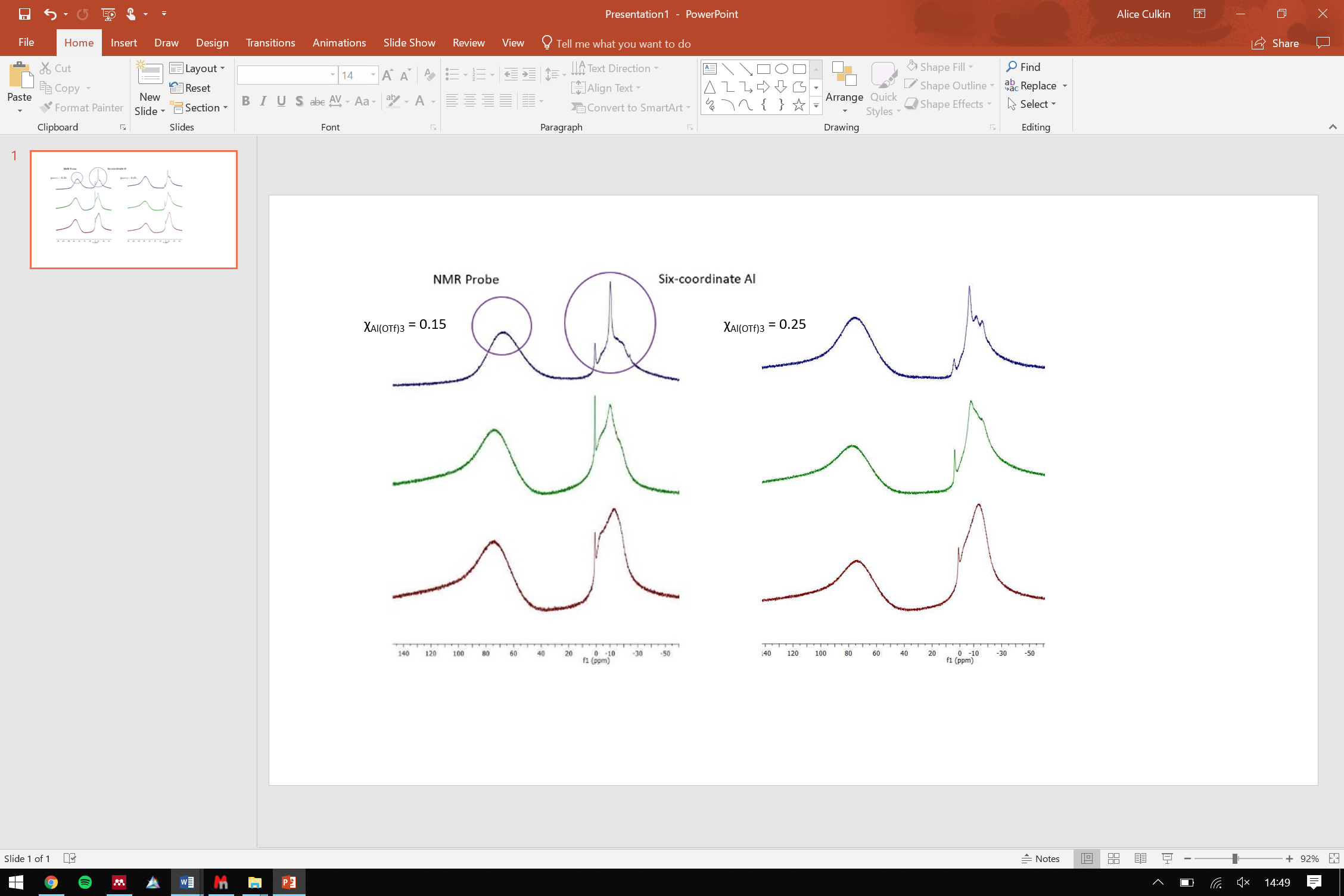


**Figure S3.** ^27^Al-NMR spectra of [C_2_mim][OTf]-Al(OTf)_3_, χ_Al(OTf)3_ = 0.15 at 27, 57 and 87 °C (neat using a DMSO capillary).


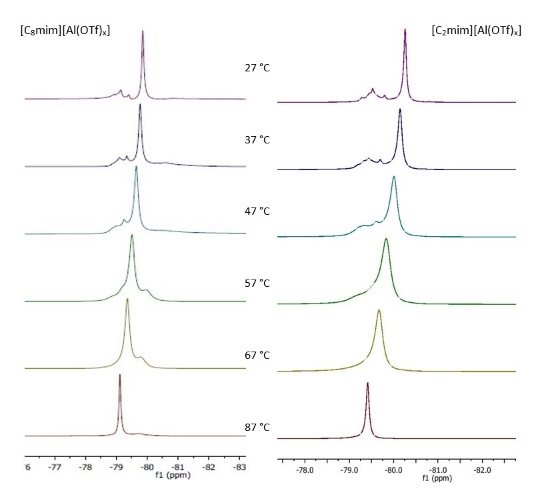


**Figure S4**. ^19^F NMR of [C_2_mim][OTf]-Al(OTf)_3_ and [C_8_mim][OTf]-Al(OTf)_3_, χ_Al(OTf)3_ = 0.15 at 27, 37, 47, 57, 67 and 87 °C (neat using a DMSO capillary).


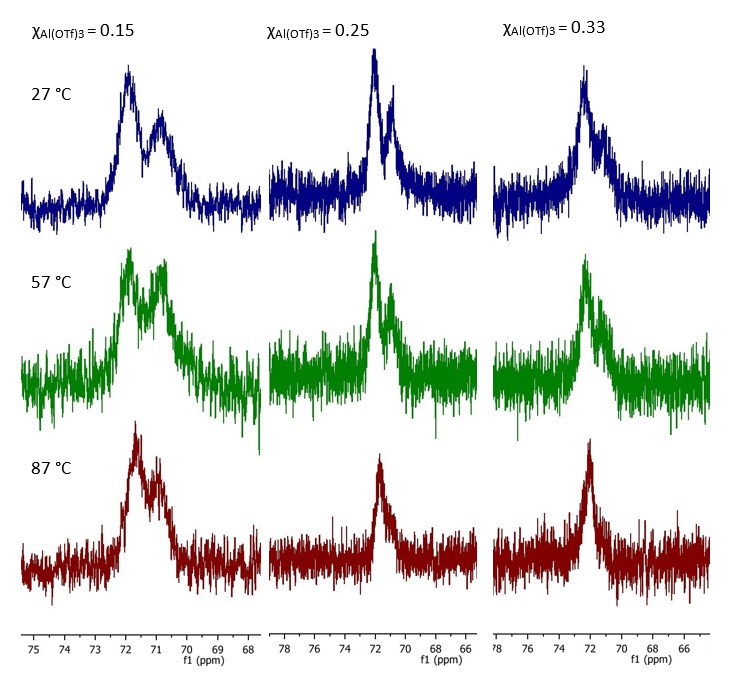


**Figure S5**. ^31^P-NMR spectra of [C_8_mim][OTf]-Al(OTf)_3_, χ_Al(OTf)3_ = 0.15, 0.25 and 0.33 at 1% TEPO at 27, 57 and 87 °C

**Figure S6**. DSC traces recorded for [C_2_mim][OTf]-Al(OTf)_3_, χ_Al(OTf)3_ = 0.15 (a) and 0.25 (b), and for [C_8_mim][OTf]-Al(OTf)_3_, χ_Al(OTf)3_ = 0.15 (c), 0.25 (d) and 0.33 (e), showing main glass transition onsets between -75.15 and -13.15 °C.

**3. Retinyl carbocation formation – UV/VIS measurements**

***Retinyl carbocation formation***. Solutions of retinyl acetate, [C_2_mim][OTf]-Al(OTf)_3_, χ_Al(OTf)3_ = 0.25, Al(OTf)_3_, La(OTf)_3_ (0.0003 M) and TfOH (0.0001 M) in nitrobenzene were prepared. The retinyl acetate solution (1.5 mL) was transferred to a quartz cuvette, 1.5 mL of the acid solution was added to the retinyl acetate mixture, and UV/Vis spectra of the mixtures were recorded using a Jasco V-650 spectrophotometer.

**
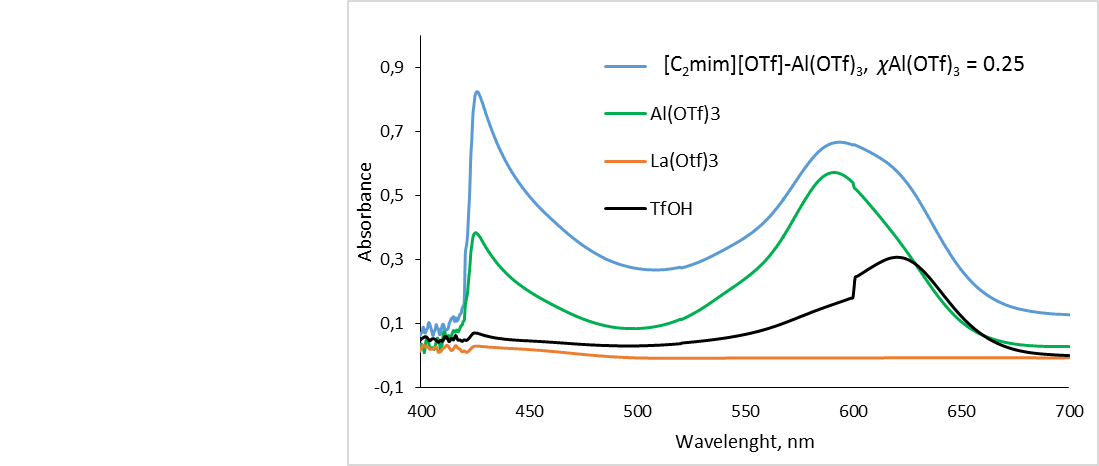
**

**Figure S7.** UV/Vis measurements.

[C_2_mim][OTf]-Al(OTf)_3_, *χ*Al(OTf)_3_ = 0.25

**3. GC-MS analysis of intermediate products**

**2,​4-​dimethyl-​1-​[(3-​methyl-​3-​buten-​1-​yl)​oxy]​-benzene:** (EI) m/z (%) 190 (25, M^+∙^), 175 (100), 160 (20), 145 (15), 115 (12), 91 (17), 77 (11), 65 (10), 41 (10), 39 (10).

**2,​4-​dimethyl-​6-​(3-​methyl-​3-​buten-​1-​yl)​phenol:** (EI) m/z (%) 190 (90, M^+∙^), 175 (100), 160 (35), 147 (27), 134 (75), 115 (25), 91 (40), 77 (30), 65 (20), 41 (19), 39 (21).

**4. TGA analysis of ionic liquids**

Step

-5,0586 %

-0,5100 mg

Left Limit

463,38 °C

Right Limit

799,40 °C

Step

-57,8578 %

-5,8330 mg

Left Limit

343,31 °C

Right Limit

463,38 °C

Step

-27,9003 %

-2,8128 mg

Left Limit

230,21 °C

Right Limit

343,31 °C

? Step

-4,5187 %

-0,4556 mg

Left Limit

112,75 °C

Right Limit

230,21 °C

DTG

%

0

20

40

60

80

°C

50

100

150

200

250

300

350

400

450

500

550

600

650

700

750

Peak

415,50 °C

Peak

294,59 °C

1/°C

-0,010

-0,008

-0,006

-0,004

-0,002

°C

50

100

150

200

250

300

350

400

450

500

550

600

650

700

750

**S**

**TA**

**R**

**e**

**SW 15.00**

**Laboratorium Analizy Termicznej: METTLER**

**Figure S8.** [C_2_mim][OTf]-Al(OTf)_3_, χ_Al(OTf)3_ = 0.25.

Step

-2,4700 %

-0,2562 mg

Residue

58,2313 %

6,0403 mg

Left Limit

452,44 °C

Right Limit

799,84 °C

Step

-24,3831 %

-2,5293 mg

Residue

60,7012 %

6,2965 mg

Left Limit

346,16 °C

Right Limit

452,44 °C

Step

-11,4813 %

-1,1910 mg

Left Limit

195,48 °C

Right Limit

346,16 °C

Step

-3,4411 %

-0,3569 mg

Left Limit

25,26 °C

Right Limit

195,48 °C

DTG

%

60

70

80

90

°C

50

100

150

200

250

300

350

400

450

500

550

600

650

700

750

Peak

420,36 °C

Peak

296,66 °C

Peak

122,49 °C

1/°C

-0,004

-0,003

-0,002

-0,001

0,000

°C

50

100

150

200

250

300

350

400

450

500

550

600

650

700

750

**S**

**TA**

**R**

**e**

**SW 15.00**

**Laboratorium Analizy Termicznej: METTLER**

**Figure S9.** CNT-[C_2_mim][OTf]-Al(OTf)_3_, χ_Al(OTf)3_ = 0.25.

Step

-1,5526 %

-0,1687 mg

Residue

79,5354 %

8,6403 mg

Left Limit

455,45 °C

Right Limit

799,62 °C

Step

-9,3265 %

-1,0132 mg

Residue

81,0879 %

8,8090 mg

Left Limit

345,62 °C

Right Limit

455,45 °C

Step

-6,4525 %

-0,7010 mg

Left Limit

182,19 °C

Right Limit

345,62 °C

? Step

-2,7351 %

-0,2971 mg

Left Limit

37,07 °C

Right Limit

182,19 °C

%

80

85

90

95

°C

50

100

150

200

250

300

350

400

450

500

550

600

650

700

750

DTG

Peak

411,99 °C

Peak

296,64 °C

1/°C

-0,0020

-0,0015

-0,0010

-0,0005

°C

50

100

150

200

250

300

350

400

450

500

550

600

650

700

750

**S**

**TA**

**R**

**e**

**SW 15.00**

**Laboratorium Analizy Termicznej: METTLER**

**Figure S10.** CNT[C_2_mim][OTf]-Al(OTf)_3_, χ_Al(OTf)3_ = 0.25 (after isolation from 1^st^ cycle, and washing).

Step

-2,4469 %

-0,2481 mg

Residue

59,2273 %

6,0051 mg

Left Limit

452,85 °C

Right Limit

799,86 °C

Step

-23,7727 %

-2,4103 mg

Residue

61,6811 %

6,2539 mg

Left Limit

344,40 °C

Right Limit

452,41 °C

Step

-10,9440 %

-1,1096 mg

Left Limit

194,49 °C

Right Limit

344,40 °C

Step

-3,5937 %

-0,3644 mg

Left Limit

25,00 °C

Right Limit

194,04 °C

DTG

%

60

70

80

90

°C

50

100

150

200

250

300

350

400

450

500

550

600

650

700

750

Peak

418,00 °C

Peak

297,01 °C

Peak

122,56 °C

1/°C

-0,004

-0,003

-0,002

-0,001

°C

50

100

150

200

250

300

350

400

450

500

550

600

650

700

750

**S**

**TA**

**R**

**e**

**SW 15.00**

**Laboratorium Analizy Termicznej: METTLER**

**Figure S11.** CNT-[C_2_mim][OTf]-Al(OTf)_3_, χ_Al(OTf)3_ = 0.25 (after regeneration).

DTG

Step

-2,0302 %

-0,2136 mg

Residue

64,7377 %

6,8114 mg

Left Limit

464,96 °C

Right Limit

799,39 °C

Step

-19,6988 %

-2,0726 mg

Residue

66,7685 %

7,0251 mg

Left Limit

344,53 °C

Right Limit

464,51 °C

Step

-9,2158 %

-0,9696 mg

Left Limit

192,93 °C

Right Limit

344,53 °C

Step

-3,8806 %

-0,4083 mg

Left Limit

38,54 °C

Right Limit

192,50 °C

%

70

80

90

°C

50

100

150

200

250

300

350

400

450

500

550

600

650

700

750

Peak

421,12 °C

Peak

296,47 °C

1/°C

-0,003

-0,002

-0,001

°C

50

100

150

200

250

300

350

400

450

500

550

600

650

700

750

**S**

**TA**

**R**

**e**

**SW 15.00**

**Laboratorium Analizy Termicznej: METTLER**

**Figure S12.** CNT-[C_2_mim][OTf]-Al(OTf)_3_, χ_Al(OTf)3_ = 0.25 (after 5^th^ cycle).

**6. ^1^H, ^13^C NMR analysis of 2,2-dimethyl-2,4-dimethylchromane**


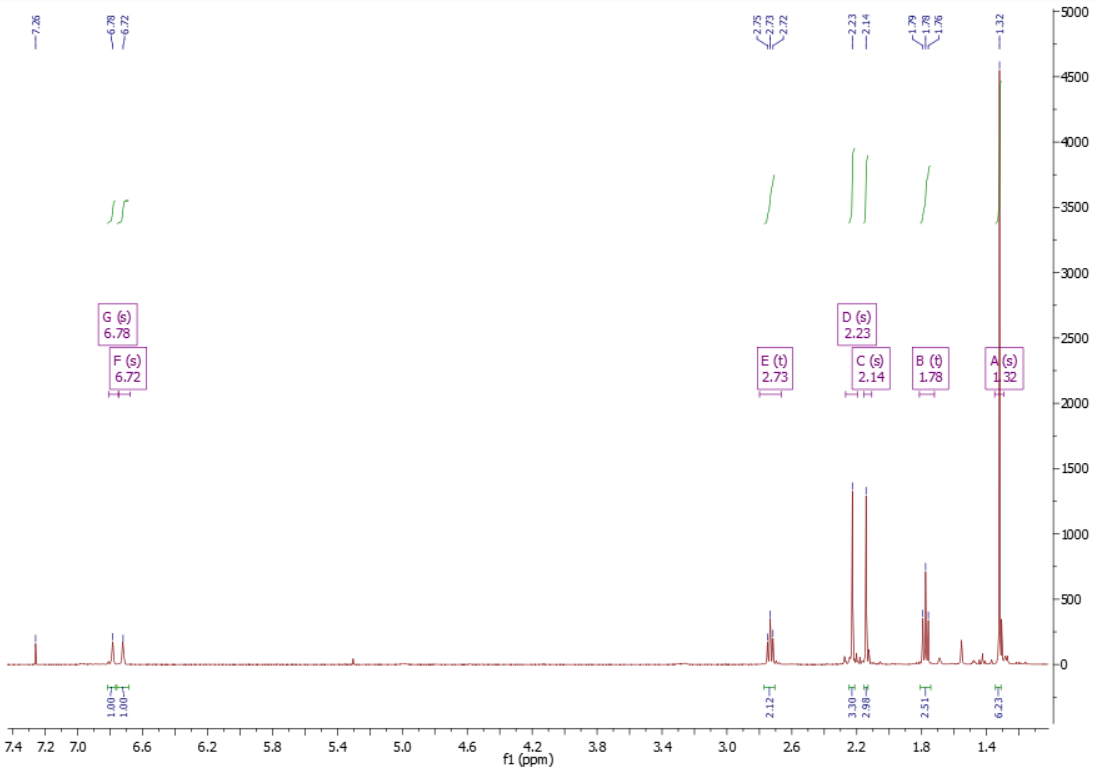

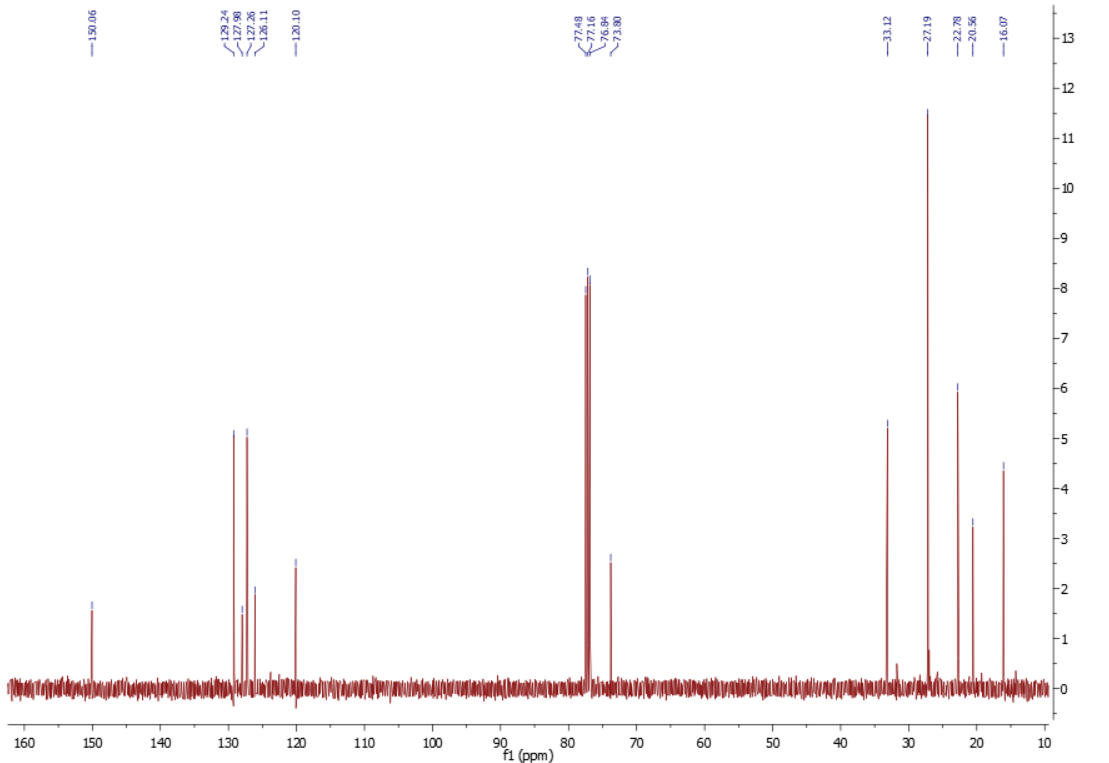

**Table S5.** Characterisation of MWCNTs support and catalyst

| **Material** | **SBET, m^2^g^‑1^** | **V_p_, cm^3^g^‑1^** | **IL loading, wt%** |
| --- | --- | --- | --- |
| CheapTubes™ MWCNTs | 91 | 0.38 | - |
| MWCNTs-[C_2_mim][OTf]-Al(OTf)_3_, *χ*_Al(OTf)3_ = 0.25 | 35 | 0.17 | 33 |
